# Network Diversity and Affect Dynamics: The Role of Personality Traits

**DOI:** 10.1371/journal.pone.0152358

**Published:** 2016-04-01

**Authors:** Aamena Alshamsi, Fabio Pianesi, Bruno Lepri, Alex Pentland, Iyad Rahwan

**Affiliations:** 1 Department of Electrical Engineering and Computer Science, Masdar Institute of Science & Technology, Abu Dhabi, United Arab Emirates; 2 Foundation Bruno Kessler, Trento, Italy; 3 Media Laboratory, Massachusetts Institute of Technology, Cambridge, Massachusetts, United States of America; Centre de Physique Théorique, FRANCE

## Abstract

People divide their time unequally among their social contacts due to time constraints and varying strength of relationships. It was found that high diversity of social communication, dividing time more evenly among social contacts, is correlated with economic well-being both at macro and micro levels. Besides economic well-being, it is not clear how the diversity of social communication is also associated with the two components of individuals’ subjective well-being, *positive* and *negative affect*. Specifically, positive affect and negative affect are two independent dimensions representing the experience (feeling) of emotions. In this paper, we investigate the relationship between the daily diversity of social communication and dynamic affect states that people experience in their daily lives. We collected two high-resolution datasets that capture affect scores via daily experience sampling surveys and social interaction through wearable sensing technologies: sociometric badges for face-to-face interaction and smart phones for mobile phone calls. We found that communication diversity correlates with desirable affect states–e.g. an increase in the positive affect state or a decrease in the negative affect state–for some personality types, but correlates with undesirable affect states for others. For example, diversity in phone calls is experienced as good by introverts, but bad by extroverts; diversity in face-to-face interaction is experienced as good by people who tend to be positive by nature (trait) but bad for people who tend to be not positive by nature. More broadly, the moderating effect of personality type on the relationship between diversity and affect was detected without any knowledge of the type of social tie or the content of communication. This provides further support for the power of unobtrusive sensing in understanding social dynamics, and in measuring the effect of potential interventions designed to improve well-being.

## Introduction

The advent of communication technologies and the ease of mobility have made our social networks more diverse. Almost on a daily basis, we contact different people from diverse backgrounds and relationships. One straightforward way to measure the diversity of our social networks is through considering the uniqueness of people who we interact with, possibly quantify it according to the number of people we communicate with (number of different social contacts) [1], types of relationships (e.g. friend and/or family) [2, 3] or backgrounds (e.g. ethnicities) [4, 5]. By using these measures, diversity in social networks appears to lead or correlate with desirable societal outcomes [6] such as better health [2, 3] and higher creativity [7], as well as with less desirable ones such as distrust, less voting and donation [4] and low performance in schools [8]. Social network diversity seems connected also with affect, namely the experience/feeling of emotions, in the context of daily interaction [9] and social (emotional, financial or informational) support from other people [10].

Nevertheless, the above concept of diversity does not consider the role that time plays in shaping our social interactions and, in particular, in having each of us allocate different time to different people (social contacts [11]). Such time-sensitive notion of social communication diversity (dividing time more or less evenly among social ties) has been shown to associate with economic well-being indices, such as economic development in communities [12] and individual’s financial status [13], as well as with individual outcomes such as study performance [8].

In this paper, we try to extend this body of work by investigating the relationships (if any) between such notion of diversity–which we will refer to by the term *diversity of social communication*–and two important components of subjective well-being: *positive* and *negative affect*. Specifically, positive and negative affect are two independent dimensions representing the self-reported or inferred experience (feeling) of emotions [14–16]. Positive affect (PA) reflects the degree of feeling “enthusiastic”, “active” and “alert”, whereas negative affect (NA) reflects “distress and not pleasurable engagement” feelings e.g. “anger, disgust, guilt, fear and nervousness”. Although positive affect and negative affect intuitively look inversely correlated, they exhibit a degree of distinction and independence from each other according to their intensities [15]. The presence (absence) of one type of affect does not entail the absence (presence) of the other; they are measured separately [17].

We chose affect as a proxy for subjective well-being because it has an impact on different aspects of people’s lives. For example, positive affect is associated with many desirable outcomes e.g. success [18], creative problem solving, [18] motivation and performance [19]. However, negative affect is associated and correlated with stress [20] and health complaints [21]. Interestingly, affect plays a role in organizations [22, 23], especially between groups [24].

What we are interested in understanding, therefore, is whether the changing diversity of social communication, measured on a daily basis, is anyhow related to the dynamics of positive and negative *affect states*, measured again on a daily basis. In psychology, *affect states* are momentary experiences (e.g. when a person feels anxious or angry at a particular moment).

A more complete picture of such relationship, however, cannot neglect the possible modulating role of different stable individual dispositions, called *traits*. Indeed, affect *states* are known to associate with positive and negative affect *traits* and the Big-Five personality *traits*[25–28] in such a way that, e.g. a person who has a high score on the positive affect trait will tend to experience higher levels of positive affect states. Personality and affect traits refer to stable characteristics of an individual, e.g. habitual patterns of behaviour and emotions. Hence, traits are relatively stable over time, different across individuals (e.g. some people are outgoing whereas others are shy), and influencing behaviour. Whether and how the different individual traits interact with the diversity of social interaction to yield daily affect states is open to investigation. This work brings affect and personality traits into the picture, aiming at clarifying the way (if any) they modulate the relationships between affect states and diversity of social communication.

Other aspects that should be considered are the possible differences due to the communication modality and the communication setting. Conceivably, face-to-face communication can impose different time limitation than, e.g. phone calls. Similarly, interactions in workplaces can impose different constraints (e.g. they are time limited and partially random) than daily spontaneous interactions with friends and relatives. Hence, this could reflect in diversity of social interaction and in the way it relates to affect states. In order to control for these possibilities, we exploited two data sets, one containing face-to-face interactions in a workplace and the other daily cell-phone calls.

The first dataset [29] contains the face-to-face interactions of 52 people who wore the sociometric badge [30] for 30 (working) days. The sociometric badges are wearable devices that are capable of detecting face-to-face interaction through embedded infrared sensors. The second dataset contains the cell-phone calls of 119 people, recorded for a period of 26 days. Daily experience sampling surveys were used in both cases to collect information about daily affect states.

The notion of diversity of social communication can be operationalized in two partially different ways. Starting from the distribution of time allocated to each contact during a given day, we can model the diversity as the distributional *uniformity*, with diversity decreasing when few social contacts consume more communication time than other contacts. In this view, diversity of social communication becomes similar to Shannon entropy [1, 12, 13]. A slightly different approach exploits the concept of *unequality*: diversity is high when every contact is allocated the same amount of time and it decreases when fewer contacts take a bigger share of the available time; such a notion can be operationalized through the Gini coefficient [31][32]. In principle, this quantity has an inverse relationship with Shannon entropy. However, this relationship breaks down when the number of social contacts is 1, as both Gini and Shannon entropy yield a zero value.

## Methods

### Data Collection

High-resolution sensors have made collecting and analyzing enormous amount of social interaction data possible [33–35], alleviating the exclusive reliance on self-reports based on people’s memory [36]. Moreover, sensors can log data at very fine time-scales [37] without interfering with people’s routines or consuming their time, making it easier to investigate short-duration phenomena. Therefore, we used two types of wearable sensors to track the social interaction of participants in two separate experiments: (1) sociometric badges and (2) smart phones. We collected also the affect states and traits of participants through surveys.

#### Sociometric Badges Dataset

**Fig 1:**
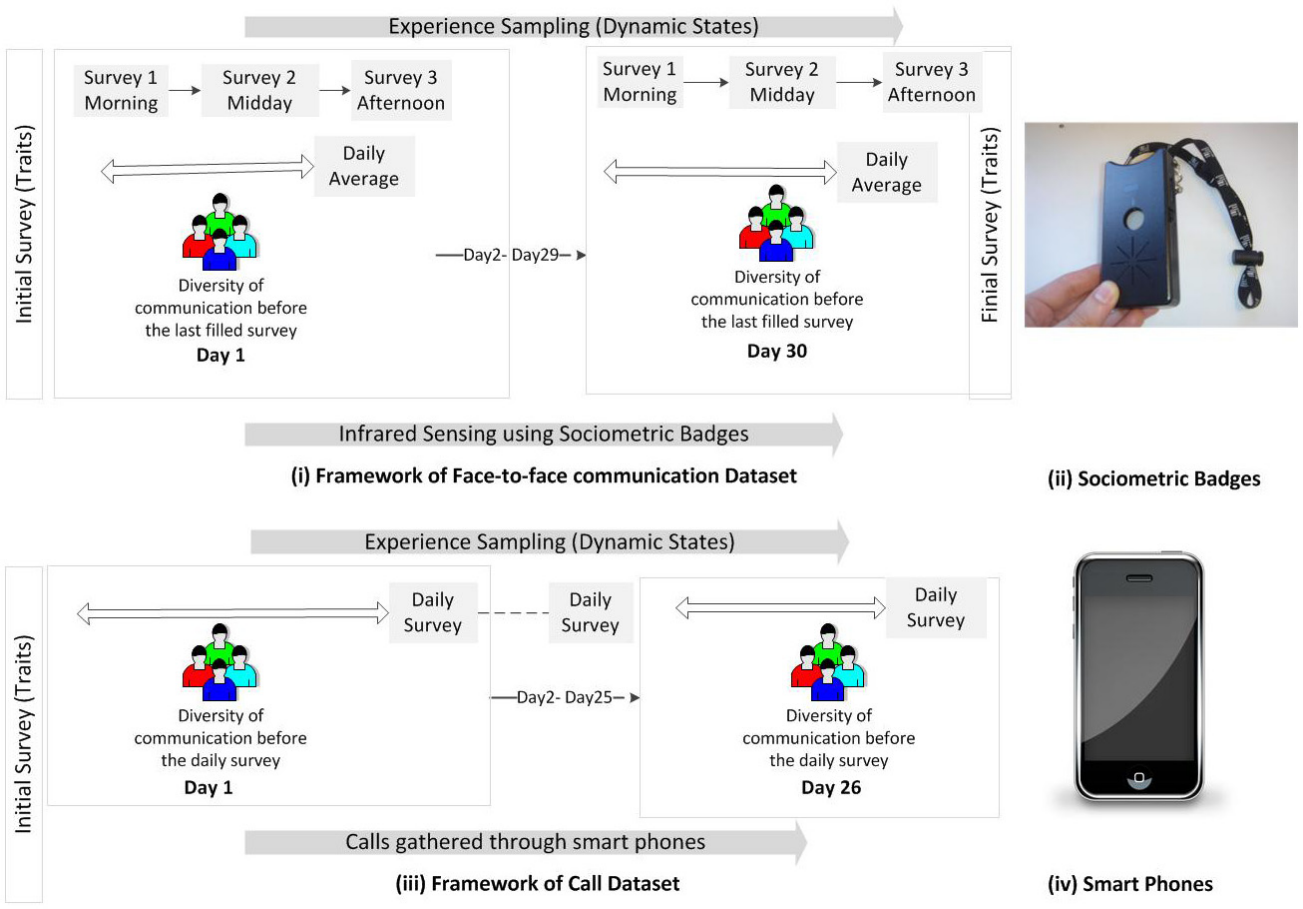
At the beginning and the end of the experiments, participants filled a survey capturing their (stable) traits. (i) Framework of the Sociometric Badges Dataset: Participants filled 3 daily surveys to measure (dynamic) states. We take the daily average of states and calculate the daily diversity in communication that took place before the last filled survey. (ii) Sociometric Badges are used to track face-to-face interactions by means of infrared (IR) sensors. (iii) Framework of the Mobile Territorial Lab project: Participants filled one daily survey to measure daily (dynamic) states. We calculate the diversity in communication that took place before the daily filled survey. (iv) Smart phones tracks the daily call social networks of participants.

*Sociometric Badges*, designed and built by author Pentland, are equipped with accelerometer, microphone, bluetooth and infrared sensors that can be used to capture (i) body movements, (ii) prosodic speech features (e.g. pitch), (iii) proximity to/colocation with other people and (iv) face-to-face interactions respectively [30]. In this work, we exploit infrared sensors –Fig 1(ii)–to recognize similar sensors facing them, implying that the two participants wearing them had a face-to-face interaction. In order for a badge to be detected through infrared sensors from another badge, the two badges must have a direct line of sight and the receiving badge’s infrared must be within the transmitting badge’s infrared signal cone of height *h* < = 1 meter and a radius of *r* < = *htanθ*, where *θ* = 15° degrees; the infrared transmission rate (TRir) was set to 1Hz.

We used these badges to track face-to-face interactions of fifty two individuals and conducted three daily experience sampling surveys [38] to collect information about their affect states: high positive affect (HPA), low positive affect (LPA), high negative affect (HNA) and low negative affect (LNA)–Fig 1(i). In addition, affect and personality (stable) traits were measured at the beginning and at the end of the study.

The study lasted 30 working days during work hours at the premises of a research organization in northern Italy. The participants are employees in a research institution in Italy who volunteered to participate in the experiment for six weeks (working days are considered only). They belong to five units whereby all the employees of these units participated in the experiment along with the heads of these units. Their ages range from 23 to 53 with an average of 36. Forty seven participants are men (90.3%) and five are women (9%). Forty seven participants are Italian (90.3%) and five participants are from other countries (9%). Forty six out of the fifty two participants were researchers in computer science belonging to four research groups; the remaining six participants were part of the full-time IT support staff. Their educational level varies from high school diplomas to PhD degrees. Following the Italian regulations, all participants were asked to sign an informed consent form and the study was conducted in accordance to the form. The general study and the form were also approved by the ethical committee of Ca’ Foscari University of Venice.

#### Procedure

The participants wore sociometric badges every working day within the institution. At the beginning and at the end of the experiment, the participants filled extended surveys about personality and affect traits. During the six weeks, participants were asked to fill three daily *experience sampling* surveys about their transient affect states that they have experienced in the last 30 minutes. It is very unlikely that people would have experienced significantly varying affect states during such a short period of time. The surveys were triggered to be sent via email every working day at 11:00 AM, 2:00 PM and 5:00 PM. The participants were given 2.5 hours to fill the surveys. We refer to the first survey as the morning survey, the second survey as the midday survey and the third survey as the afternoon survey.

Experience sampling surveys elicit dynamic states of affect. Questions in these surveys report participants’ states which were experienced in the last 30 minutes. The short version of Positive and Negative Affect Schedule (PANAS) was used to evaluate the affect states of participants [39]. Specifically, high positive affect (HPA) was assessed using 3 items: *enthusiastic*, *interested* and *active*. High negative affect (HNA) was assessed using 3 items: *sad*, *bored* and *sluggish*. Low positive affect (LPA) was assessed using 2 items: *calm* and *relaxed*, while low negative affect (LNA) was assessed using 2 items *lonely* and *isolated*. Respondents were asked to report a number on a scale from 1 to 5 (1 = *Very Slightly* or *Not At All* and 5 = *Extremely*) for each item. Then, we averaged the numbers assigned to the items describing a given state (e.g. *enthusiastic*, *interested* and *active* for HPA)

Note that the experience sampling method has a long history and is highly reliable in measuring dynamics of psychological states within individuals [40]. For those interested in the caveats around the use of experience sampling, we also point to extensive discussions elsewhere [41].

On the other hand, the Italian version of the Big Five Marker Scale (BFMS) [42] was used to assess personality traits at the beginning and at the end of the experiment. This scale is an adjective list composed by 50 items. Our sample was composed of almost 90% Italian native speakers and the subjects who were not Italian native speakers received a validated translation of the BFMS. Similarly, Multidimensional Personality Questionnaire (MPQ) was utilized to measure the affect traits of our participants [43].

#### Preprocessing the surveys

We take the average of the daily scores of dynamic affect states of each participant to associate it with the daily diversity in communication that take place before the last filled survey.

#### Dispositional Traits of Personality and Affect

We considered the trait scores that were reported by participants at the end of the experiment. Then, we normalized the trait scores of participants using the mean and the standard deviation. To discuss the statistical interaction between traits and social-situational factors associated with a given transition, we focused only on participants with high scores in the trait (+1 standard deviation) and participants with low scores in the trait (-1 standard deviation). By using this method, we are able to know how levels of traits moderates the association between the social-situational factors and the variability in states. For example, we are interested to know how introverts respond to an increase in the diversity in social communication in comparison to extroverts’ response to the same increase.

#### Mobile Territorial Lab Dataset

Mobile phones allow for unobtrusive and cost-efficient access to huge streams of previously inaccessible data related to daily social behavior [44–47]. Recently, the social psychologist Miller wrote “The Smartphone Psychology Manifesto” in which he argued that the smartphones should be seriously considered as new research tools for psychology. In his opinion, these tools could revolutionize all fields of psychology and other behavioural sciences making these disciplines more powerful, sophisticated, and grounded in real-world behaviour [48]. Indeed, several works have started using smartphone activity data in order to predict personality traits [49–51], daily mood [52] and stress levels [53].

#### Procedure

We leveraged the sensing technologies that are available in smart phones and tracked the daily call social networks of 119 participants–the interlocutors could be within or outside the community of participants–in Trento, Italy for 26 days. Simultaneously, daily experience sampling surveys were conducted to collect two affect states and their corresponding traits–high positive affect (HPA) and high negative affect (HNA)–Fig 1(iii). At the beginning of the study affect and personality (stable) traits were measured.

The study was conducted within the Mobile Territorial Lab (http://www.mobileterritoriallab.eu), a joint initiative created by Telecom Italia, Fondazione Bruno Kessler, MIT Media Lab and Telefonica [54]. Following the Italian regulations, all participants were asked to sign an informed consent form and the study was conducted in accordance to it. The general study and the form were also approved by a joint Ethical Committee of University of Trento and Province of Trento (for more details about Mobile Territorial Lab, check S1 File).

A total of 119 volunteers from the MTL chose to participate in our data collection of daily affect states. Their ages range from 28 to 50 with an average of 39. Forty three participants are men (36%) and seventy six are women (63%). 115 participants are Italian (96.6%) and four participants are from other countries (3.4%). They held a variety of occupations and education levels, ranging from high school diplomas to PhD degrees. All were savvy Android users who had used the smartphones provided by the MTL since November 2012.

During the 26 days, participants were asked to fill daily experience sampling surveys about transient affect states that they experience. The surveys were triggered to be sent via email and via SMS every day at 8:00 PM and the participants were given four hours to fill the surveys.

The short version of Positive and Negative Affect Schedule (PANAS) was used to evaluate the affective daily states of participants [39]. Specifically, positive affect (PA) was assessed using 5 items: *alert*, *inspired*, *determined*, *attentive* and *active*. Negative affect (NA) was assessed using 5 items: *upset*, *hostile*, *ashamed*, *nervous* and *afraid*.

At the beginning of the study, the participants filled extended surveys about personality and affect traits. The long version of Positive and Negative Affect Schedule (PANAS) was used to measure the affect traits’ scores of our participants [14].

#### Preprocessing the surveys

The data comprise 1499 surveys by the 119 participants. Ideally, the number of filled surveys should be 3094 (119 participants × 1 daily surveys × 26 days). However, participants sometimes do not fill the daily surveys or fill them late. Because we aimed at relating the affect states to the preceding diversity in communication, the time window of the preceding communication is about twenty four hours approximately. Therefore, many surveys were not taken into consideration. Regarding the affect states, we took the exact daily reported scores.

#### Dispositional Traits of Personality and Affect

We followed the same described approach for the sociometric badges dataset in Section “Sociometric Badges Dataset”.

### Dynamic Social networks for both Datasets

We created dynamic temporal networks of face-to-face interaction and mobile phone communication for each participant in the two datasets. For each day, we created the participant’s temporal dynamic social network based on the social ties that the infrared sensor has detected or the phone calls that the participant has received or initiated. We considered only social networks that took place before the last filled daily survey.

Due to the difference in the types of the communication, the scope of the networks in the two types differ too. The sociometric badges detect face-to-face interaction between participants only, while smart phones can track calls between participants and other people–participants or non-participants.

**Fig 2:**
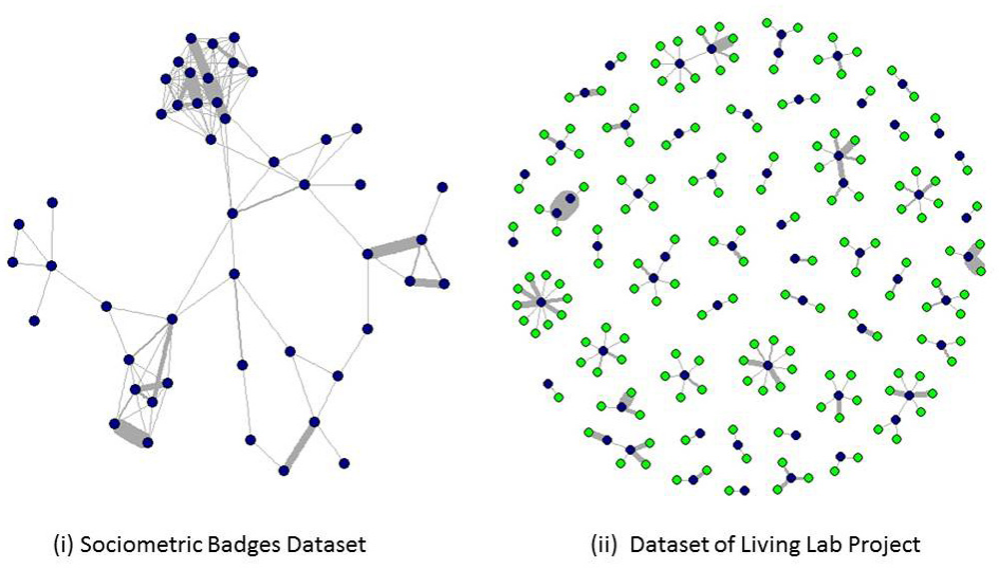
Sample daily networks of the two datasets. (i) The network of the community of participants is constructed based on the sociometric badges dataset in one typical day. The infrared sensors can only detect the infrared sensors of other participants, so the network includes face-to-face interactions within the community of participants. (ii) The call network is constructed including people within or outside the participant community in one typical day. MTL participants are coloured with dark blue while non-participants are coloured with green. In both networks, the thickness of edges represents the intensities of communication between two nodes. We can see that individuals are inclined to divide their time unequally among their social contacts.

**Fig 3:**
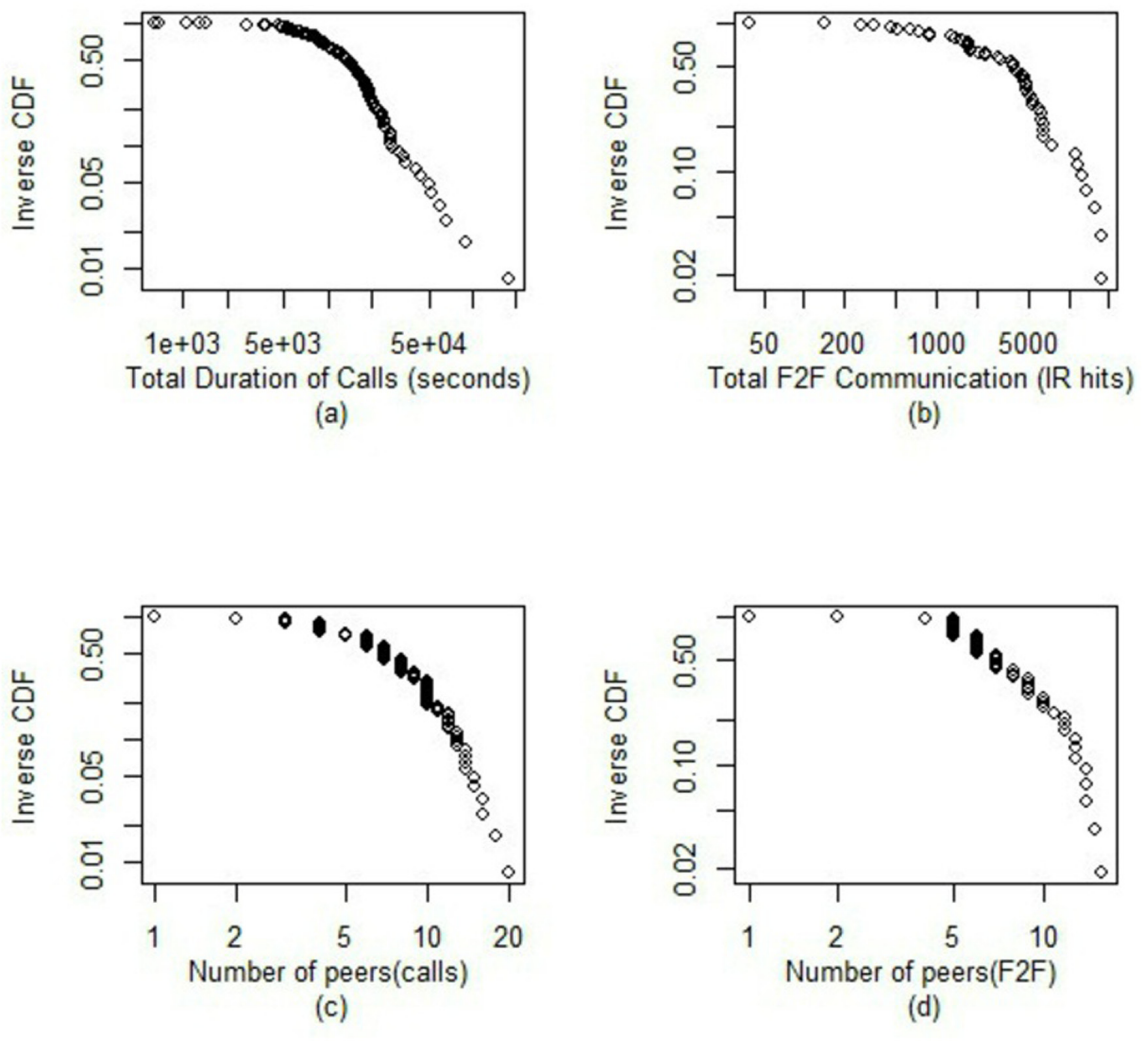
(a) Distribution of total time spent on phone calls by participants (b) Distribution of total time spent communicating through face-to-face interaction (c) Distribution of personal network sizes via mobile phones calls (d) Distribution of personal network sizes via face-to-face interaction. Generally, few individuals have very long phone calls or face-to-face interactions. Also, few individuals have a high number of social contacts.

The dynamic social networks of the two datasets in a particular day are depicted in Fig 2. Fig 3 show the distribution of aggregate communication and social network sizes of participants in the two datasets.

### Diversity

#### Diversity as Distributional Uniformity

One way of modeling the diversity of social interaction is through using the notion of distributional *uniformity*, with: a) diversity is high when all social contacts receive the same amount of interaction time and b) diversity decreases when fewer social contacts receive more interaction time than other social contacts. By measuring the uncertainty of a random variable, Shannon entropy is a good summary measure of distributional uniformity and for this reason, it has been exploited to address diversity [1, 12, 13].

Entropy is adapted to suite interaction networks by first calculating, for node (person) *i*, the share of communication time (*p*_*ij*_) he/she has shared with each of his/her interacting partners (other nodes) *j*, using Formula 1 where: *V*_*ij*_ is the communication volume between node i and j (number of infrared contacts in face-to-face communication or the total call duration).

$$\begin{array}{c} p_{ij}=\frac{V_{ij}}{\sum_{j=1}^{k}V_{ij}} \end{array}$$ (1)

*p*_*ij*_ is then plugged in Formula 2 where k is the degree of node i (the number of i’s contacts) and *p*_*ij*_ is the proportion of i’s total communication volume that involves j, normalized by *log*_2_(*k*).

$$\begin{array}{c} D_{social}\left(i\right)=-\frac{-\sum_{j=1}^{k}p_{ij}log_{2}\left(p_{ij}\right)}{log_{2}\left(k\right)} \end{array}$$ (2)

#### Diversity as inequality

If we assume that a given person, on a given day, has a given amount of time to distribute among his/her (potential) social contacts, then the diversity of social interaction comes close to *inequality*. This quantity is low when every contact is allocated the same amount of time and it increases as soon as fewer contacts take a bigger share of the available time. When seen from this perspective, the diversity of social interaction can be operationalized through the Gini coefficient, whose typical application has been the measurement of the inequality of income distribution in a given population [32]

We used Formula 3 to compute the Gini coefficient, where *k* is the number of contacts and *V*_*ij*_ is the volume of communication that involves *j*.

$$\begin{array}{c} D_{social}\left(i\right)=\frac{2\sum_{j=1}^{k}jV_{ij}}{k\sum_{j=1}^{k}V_{ij}}-\frac{k+1}{k} \end{array}$$ (3)

Gini coefficient will be zero (no inequality) in two cases: a) when there are n > 1 contacts and the communication is equally distributed among them and b) when there is just one contact who takes all the communication time. The latter case fully accords with the view of diversity as *inequality*: as there are no *competing* contacts, there is no inequality. In principle, Gini has an inverse relationship with Shannon entropy. This relationship breaks down when the number of contacts is 1, as both Gini and Shannon entropy yield a zero value (no uncertainty = no uniformity but no inequality). For more details, we compare the behaviour of each diversity index in different scenarios in S1 File.

## Results

We first describe the statistical model that we used. Then, we present our results and discuss them.

### Statistical Model

Both our independent variables (levels of the various traits and diversity measures–either Shannon entropy or Gini coefficient) and dependent variables (HPA, LPA, HNA and LNA-state levels) are continuous. Hence, we used linear regression to model the relationship between dependent and independent variables. As both datasets contain longitudinal data, with repeated measures, OLS-based models are not an appropriate option, given that they do not capture within-subject correlation. We therefore used linear mixed models that allow for a flexible modeling of within-subject dependencies through different choices of co-variance structures. Other advantages of exploiting linear mixed models are: the inclusion of both random and fixed effects–that allows for to consider subjects as a random sample from the population– and higher robustness to unbalanced longitudinal data set with respect to repeated measure ANOVA.

#### Goodness of fit

It is essential to measure how well our model performs. Hence, we used the variance explained (*R*^2^) but its calculation is not straightforward in mixed models. Recently, Nakagawa and Schielzeth proposed two types of *variance explained* *R*^2^: (1) marginal *R*^2^ and (2) conditional *R*^2^ [55]. Marginal *R*^2^ addresses the variance explained by fixed effects, while the conditional *R*^2^ addresses the variance explained by fixed and random effects together. We used their method to calculate the *R*^2^. In our case, the marginal *R*^2^ (addressing the variance explained by fixed effects) is more relevant to quantify the goodness of model fit.

### The role of diversity

**Fig 4:**
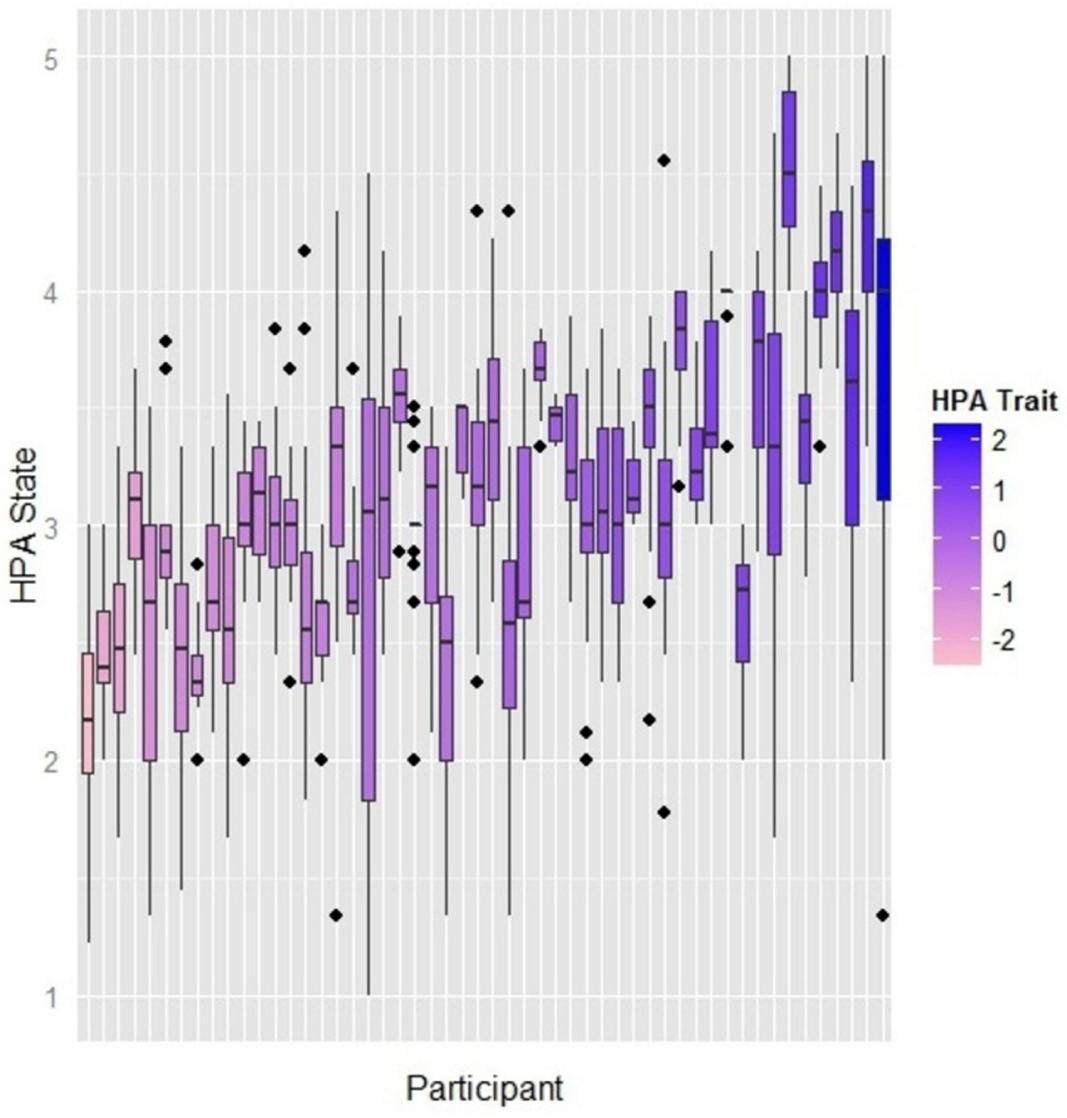
Distribution of dynamic HPA state by participant and trait in the sociometric badges dataset. Each boxplot describes the distribution of the high positive affect (HPA) state score for each participant. The colour intensity indicates the individual’s score of the corresponding trait (HPA trait). The dark blue colour means that a participant has a low score in the HPA trait, while the light blue colour means that a participant has a high score in the HPA trait. The boxplots are sorted in an increasing order according to the score of the HPA trait. Generally, people who have high scores in the trait (darker colours) tend to have high scores in the scores of the corresponding state as well. The same applies to people who have low scores in the trait (lighter colours) who tend to have low scores in the corresponding state as well. However, there are many exceptions whereby the dispositional trait of HPA do not explain the daily scores of the state.

In the context of face-to-face interactions, we analyzed 912 daily records of 52 participants and addressed four affect state: high and low positive affect (HPA/LPA) along with high and low negative affect (HNA/LNA). In phone call networks, we analyzed 1499 daily records of 119 participants and addressed two affect states: high positive affect (HPA) and high negative affect (HNA). Fig 4 depicts the within-subject variations in the HPA dynamic state and the between-subject variations in the HPA stable trait. The trait can partially explain the accompanying scores of the dynamic states. Nevertheless, there is a lot of variations that traits alone do not seem to be able to explain that are expected to exist due to situational factors. In this case, we consider the diversity of social communication as a situational factor that can explain some of the variability in the affect states.

We started with our basic model that consists of a diversity measure: entropy or Gini, (*X*_*ij*_) that captures its fixed and random effects. *X*_*ij*_ is the diversity of the social communication of participant *i* at day *j*. Let *Y*_*ij*_ denotes the addressed affect state of participant *i* at day *j*. For each affect state and communication type (face-to-face or phone calls), we fit the model in Formula 4 where *β*_0_ is a constant (intercept), *β*_1_ is the fixed effect slope of the used diversity measure, *β*_2_ is the random slope of the used diversity measure, *u*_*j*_ is the fixed intercept of individuals and *e*_*ij*_ is the time-specific error of participant *i* at time *j*.

$$\begin{array}{c} Y_{ij}=\beta_{0}+\beta_{1}X_{ij}+\beta_{2}X_{ij}+u_{j}+e_{ij} \end{array}$$ (4)

According to our results, we found that the role of diversity in social communication is not present in all settings, e.g. types of communication and affect states. In some cases, the two diversity measures (Gini and entropy) gave statistically significant results. However, in these cases, the direction of the relationship between the diversity and the affect state is not consistent. Moreover, the *R*^2^ (variance explained of models containing only diversity measures) is very small (< 0.1). See S1 File for details. Therefore, the relationship between diversity in communication and affect dynamic states is weak and insufficient alone to explain the dynamics of daily affect states.

### Individual Differences

Motivated by the fact that personality traits can partially explain the variability in affect states [27, 28, 56]–it is also observed in Fig 4–especially when the traits are supported by situational factors [26, 57], we hypothesize that the interplay between the trait and the diversity of communication (the situational factor) can better explain affect dynamics.

**Fig 5:**
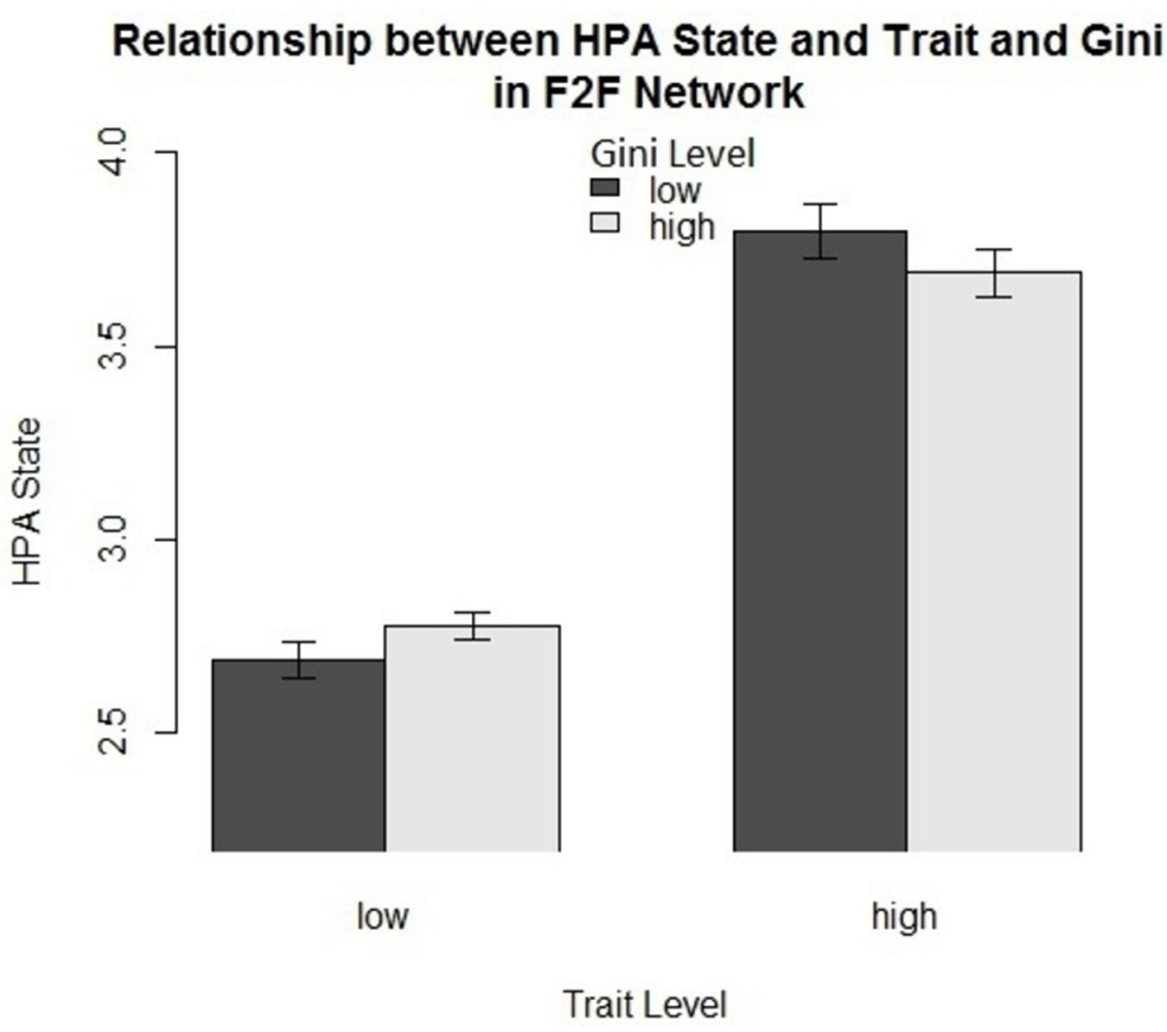
The bar plot demonstrates the mediating effect of traits in the relationship between the diversity measure and the score of the dynamic affect state. Generally, high scores of the trait correspond to high scores of the state. However, if we look for within-trait variation of dynamic state, we can see that diversity plays different roles in different levels of the trait. For example, when the trait level is low, the score of the state is relatively high for high scores of Gini and low for low scores of Gini. In contrast, when the trait level is high, the score of the state is relatively high for low scores of Gini and low for high scores of Gini.

We turn now to consider the role (if any) played by the traits. We started with a visual inspection by plotting the interaction bar plot of the affect states, broken down by traits and diversity measures. Fig 5 shows the relationship between the level of positive affect *trait* (x-axis), the score of the positive affect *state* (y-axis) and the discretized level of the Gini score–black bars represent low scores of Gini and gray bars represent high scores of Gini. We discretized the values of the trait and the diversity measure (high and low) to demonstrate the role of the interaction between the trait and the diversity measure in affect dynamics. Obviously, the affect *state* scores correspond to the affect *trait* scores. In other words, people with high scores in the trait almost experience high scores of the affect state as well. However, when we consider the Gini coefficient, one can notice the effect of the trait score on the state score depends on the level of Gini score. When the trait is high, the mean score of the state is relatively low when Gini is high, while the mean score of the state is relatively high when Gini is low. The opposite can be witnessed in the case of low trait scores. Hence, the plot suggests that traits might indeed play a role in mediating the relationship between diversity in social communication and affect states. The purpose of the visualization is illustrating the suggested role of the interaction between traits and diversity measures in one example. Nevertheless, not all results are this clearly visible because the data is based on longitudinal studies, in which addressed variables (diversity and affect state) are sampled many times for each participant.

This visualization is giving us a hint that the role of diversity in affect dynamics is mediated by traits. However, we need to affirm the significance of the initial observations statistically using an appropriate statistical tool for longitudinal data. Therefore, we used the complete model that contains all the variables in the basic model plus the chosen individual trait (*T*_*i*_) of participant *i* and the interaction between the trait and the diversity measure (*T*_*i*_ * *X*_*ij*_) where *β*_3_ is the fixed effect of the trait and *β*_4_ is the fixed effect of the interaction between the trait and the diversity measure in Formula 5.

$$\begin{array}{c} Y_{ij}=\beta_{0}+\beta_{1}X_{ij}+\beta_{2}X_{ij}+\beta_{3}T_{i}+\beta_{4}T_{i}*X_{ij}+u_{j}+e_{ij} \end{array}$$ (5)

#### Deviance Test

In addition to considering the significance of the fixed effect parameters, it is important to also investigate whether the more complex model indeed provides a better fit to the data. To this end, we exploit the *deviance test* which compares the deviances of the two models (the negative of twice the log-likelihood of each model) and checks whether the more complex one has a significantly lower deviance than the basic one. If so, we can confidently conclude that the more complex model yields a better fit to the data. Otherwise, the basic model is preferred.

We found that the role of diversity of communication is dependent on the addressed affect state, the psychological trait, the selected diversity measure and the communication type. For instance, in the context of face-to-face interaction, diversity (calculated by Gini) correlates negatively with high positive affect (HPA) for individuals who have low scores in the corresponding HPA, while it correlates positively with HPA state for individuals who have high scores in the HPA trait (Marginal *R*^2^ = 0.3 and pr(*χ*^2^) = 0.03). In the context of call networks, however, the diversity, measured by Gini, correlates positively with HPA for introverts and vice versa for extroverts (Marginal *R*^2^ = 0.013 and pr(*χ*^2^) = 0.06). We can see that the moderating trait differs according to the mode of communication in the case of HPA. In other words, it is not only the case that traits can moderate the effect of situational aspects (diversity in communication), but also the moderating trait and the direction of the interaction can vary according to the scenario (face-to-face vs phone call). In the context of calls, we found also that diversity, measured by entropy, correlates positively with high negative affect (HNA) for individuals who have low scores in the corresponding trait, while diversity correlates negatively with HNA for people with low scores in HNA trait (Marginal *R*^2^ = 0.11 and pr(*χ*^2^) = 0.03). According to the results, the complete model outperforms the basic model in most cases–as measured by *χ*^2^ test. Said differently, the additional variance explained by the interaction of the diversity measures and traits is statistically significant in comparison to the models that include the traits only. For more details, check S1 File.

## Discussion

Methodologically, our work demonstrates how to dynamically capture common types of social communication, face-to-face communication and phone calls, and link them to affect dynamics using automated sensors and experience sampling respectively. Our methodology can be adopted by similar studies that aim at relating social communication with dynamic characteristics and behaviours. Through our methodology, we showed that despite using minimal features of interaction–without the need to know the time or content of conversations, even the types of social relationship–we can still predict variation in affect states. We relied merely on pure structural properties of dynamic social networks of individuals, specifically diversity of social communication, and their personality traits to infer scores of affect states. With such a non-intrusive approach, we were able to delineate the relationship between diversity of social communication and subjective well-being.

While network diversity was directly correlated with economic well-being in earlier studies, we found that the association between network diversity and subjective well-being is dependent on individual differences and communication modes. Specifically, we found that diversity of communication is beneficial for certain personality types in some communication types, while it is not beneficial for other personality types. For example, diversity in phone calls is experienced as good by introverts, but bad by extroverts; diversity in face-to-face interaction is experienced as good by people who tend to be positive by nature (trait) but bad for people who tend to be not positive by nature. Our findings contribute to the growing literature in social psychology that focuses on understanding the role of situational factors in the variability of our affect. Also, our findings bring attention to the role of individual differences (personalities) and the necessity of considering them while studying affect dynamics.

Interestingly, our results suggest also that the *diversity as inequality* (Gini) captures the relationship between diversity in social communication and affect dynamics better than the *diversity as uniformity* (entropy), in particular in the face-to-face interaction scenario. Further investigation is required to confirm this result.

Although our study is a pioneer in studying the relationship between affect dynamics and diversity in social communication, the nature of the observational studies from which the datasets were collected, restricts our conclusions to be correlational rather than causal. The confirmation of the causality of the results requires conducting controlled experiments in which different interventions should be introduced to different groups of people in order to elicit the causal effect of diversity of social communication.

In this study, we focused only on direct communication: phone calls and face-to-face communication. Nowadays, people do not only communicate through these modes, but also use social media and instant messaging heavily. Therefore, it would be interesting to study the relationship between diversity of social communication through these passive types of communication and affect dynamics.

## Supporting Information

S1 File The file contains the following. Sections: 1 Sociometric Badges Dataset, 2 Mobile Territorial Lab Dataset, 3 Dynamic Social networks, 4 Diversity Measures, 5 Statistical Models, 6 Results. Table 1, Surveys for personality and affect states and traits in the Sociometric Badges study. Tables 2:28, statistical results. Figure 1, Descriptive statistics of the participants in the Sociometric Badges Dataset. Figure 2, Descriptive statistics of the participants in the Mobile Territorial Lab Dataset. Figure 3, The composite social network of participants in the sociometric badges dataset. Figure 4, The composite social network of participants in the MTL dataset. Figure 5, The daily social networks of the participants in the sociometric badges (face to face interaction) for days 1–15. Figure 6, The daily social networks of the participants in the sociometric badges (face to face interaction) for days 16–30. Figure 7, The daily social networks of the participants in the Living Lab Dataset (mobile phone communication) for days 1–15. Figure 8, The daily social networks of the participants in the Living Lab Dataset (mobile phone communication) for days 16–26. Figure 9, The entropy is plotted against different probabilities of a random variable. The random variable can take only two values (Bernoulli Process). Figure 10, The Gini is plotted against different probabilities of a random variable. The random variable can take only two values (Bernoulli Process).
(PDF)

S1 Datasets Datasets of face-to-face interactions and mobile phone communication. (RAR)

## References

[1] Madan A, Cebrian M, Lazer D, Pentland A. Social sensing for epidemiological behavior change In: Proceedings of the 12th ACM international conference on Ubiquitous computing. ACM; 2010 p. 291–300. 10.1145/1864349.1864394

[2] Cohen S, Doyle WJ, Skoner DP, Rabin BS, Gwaltney JM. Social ties and susceptibility to the common cold. Jama. 1997;277(24):1940–1944. 9200634

[3] Cohen S, Janicki-Deverts D. Can we improve our physical health by altering our social networks? Perspectives on Psychological Science. 2009;4(4):375–378. 10.1111/j.1745-6924.2009.01141.x 20161087 PMC2744289

[4] Putnam RD. E pluribus unum: Diversity and community in the twenty-first century the 2006 Johan Skytte Prize Lecture. Scandinavian political studies. 2007;30(2):137–174. 10.1111/j.1467-9477.2007.00176.x

[5] Villalpando O. The impact of diversity and multiculturalism on all students: Findings from a national study. Journal of Student Affairs Research and Practice. 2002;40(1):124–144. 10.2202/1949-6605.1194

[6] Granovetter MS. The strength of weak ties. American journal of sociology. 1973;p. 1360–1380. 10.1086/225469

[7] Perry-Smith JE. Social yet creative: The role of social relationships in facilitating individual creativity. Academy of Management journal. 2006;49(1):85–101. 10.5465/AMJ.2006.20785503

[8] Vaquero LM, Cebrian M. The rich club phenomenon in the classroom. Scientific reports. 2013;3 10.1038/srep01174 23378908 PMC3558720

[9] Sandstrom GM, Dunn EW. Social Interactions and Well-Being The Surprising Power of Weak Ties. Personality and Social Psychology Bulletin. 2014;p. 0146167214529799. 10.1177/0146167214529799 24769739

[10] Agneessens F, Waege H, Lievens J. Diversity in social support by role relations: A typology. Social networks. 2006;28(4):427–441. 10.1016/j.socnet.2005.10.001

[11] Miritello G, Moro E, Lara R, Martínez-López R, Belchamber J, Roberts SG, et al Time as a limited resource: Communication strategy in mobile phone networks. Social Networks. 2013;35(1):89–95. 10.1016/j.socnet.2013.01.003

[12] Eagle N, Macy M, Claxton R. Network diversity and economic development. Science. 2010;328(5981):1029–1031. 10.1126/science.1186605 20489022

[13] Pan W, Aharony N, Pentland A. Fortune monitor or fortune teller: Understanding the connection between interaction patterns and financial status In: Privacy, Security, Risk and Trust (PASSAT) and 2011 IEEE Third Inernational Conference on Social Computing (SocialCom), 2011 IEEE Third International Conference on. IEEE; 2011 p. 200–207. 10.1109/PASSAT/SocialCom.2011.163

[14] Watson D, Clark LA, Tellegen A. Development and validation of brief measures of positive and negative affect: the PANAS scales. Journal of personality and social psychology. 1988;54(6):1063 10.1037/0022-3514.54.6.1063 3397865

[15] Diener E, Larsen RJ, Levine S, Emmons RA. Intensity and frequency: dimensions underlying positive and negative affect. Journal of personality and social psychology. 1985;48(5):1253 10.1037/0022-3514.48.5.1253 3998989

[16] Russell J. A circumplex of affect. Journal of Personality and Social Psychology. 1980;36:1152–1168.

[17] Lucas, RE, Dyrenforth PS. Does the existence of social relationships matter for subjective well-being? 2006;.

[18] Lyubomirsky S, King L, Diener E. The benefits of frequent positive affect: does happiness lead to success? Psychological bulletin. 2005;131(6):803 16351326 10.1037/0033-2909.131.6.803

[19] Erez A, Isen AM. The influence of positive affect on the components of expectancy motivation. Journal of Applied psychology. 2002;87(6):1055 10.1037/0021-9010.87.6.1055 12558213

[20] Clark L, Watson D. Diurnal variation in mood: Interaction with daily events and personality In: meeting of the American Psychological Association, Washington, DC; 1986.

[21] Beiser M. Components and correlates of mental well-being. Journal of Health and Social Behavior. 1974;p. 320–327. 10.2307/2137092 4455735

[22] Barsade S, Brief AP, Spataro SE, Greenberg J. The affective revolution in organizational behavior: The emergence of a paradigm. Organizational behavior: A management challenge. 2003;1:3–50.

[23] Barsade SG, Gibson DE. Why does affect matter in organizations? The Academy of Management Perspectives. 2007;21(1):36–59. 10.5465/AMP.2007.24286163

[24] Barsade SG, Ward AJ, Turner JD, Sonnenfeld JA. To your heart’s content: A model of affective diversity in top management teams. Administrative Science Quarterly. 2000;45(4):802–836. 10.2307/2667020

[25] Costa PT, McCrae RR. The revised neo personality inventory (neo-pi-r). The SAGE handbook of personality theory and assessment. 2008;2:179–198.

[26] Zelenski JM, Larsen RJ. The distribution of basic emotions in everyday life: A state and trait perspective from experience sampling data. Journal of Research in Personality. 2000;34(2):178–197. 10.1006/jrpe.1999.2275

[27] Tellegen A. Structures of mood and personality and their relevance to assessing anxiety, with an emphasis on self-report. 1985;.

[28] Watson D, Clark LA. Negative affectivity: the disposition to experience aversive emotional states. Psychological bulletin. 1984;96(3):465 10.1037/0033-2909.96.3.465 6393179

[29] Lepri B, Staiano J, Rigato G, Kalimeri K, Finnerty A, Pianesi F, et al The sociometric badges corpus: A multilevel behavioral dataset for social behavior in complex organizations In: Privacy, Security, Risk and Trust (PASSAT), 2012 International Conference on and 2012 International Confernece on Social Computing (SocialCom). IEEE; 2012 p. 623–628.

[30] Olguín DO, Waber BN, Kim T, Mohan A, Ara K, Pentland A. Sensible organizations: Technology and methodology for automatically measuring organizational behavior. Systems, Man, and Cybernetics, Part B: Cybernetics, IEEE Transactions on. 2009;39(1):43–55. 10.1109/TSMCB.2008.2006638 19150759

[31] Gini C. Variabilità e mutabilità Reprinted in Memorie di metodologica statistica (Ed Pizetti E, Salvemini T) Rome: Libreria Eredi Virgilio Veschi 1912;1.

[32] Yitzhaki S. Relative deprivation and the Gini coefficient. The quarterly journal of economics. 1979;p. 321–324. 10.2307/1883197

[33] Cattuto C, Van den Broeck W, Barrat A, Colizza V, Pinton JF, Vespignani A. Dynamics of person-to-person interactions from distributed RFID sensor networks. PloS one. 2010;5(7):e11596 10.1371/journal.pone.0011596 20657651 PMC2904704

[34] Stehlé J, Voirin N, Barrat A, Cattuto C, Isella L, Pinton JF, et al High-resolution measurements of face-to-face contact patterns in a primary school. 2011;. 10.1371/journal.pone.0023176 PMC3156713 21858018

[35] Aharony N, Pan W, Ip C, Khayal I, Pentland A. Social fMRI: Investigating and shaping social mechanisms in the real world. Pervasive and Mobile Computing. 2011;7(6):643–659. 10.1016/j.pmcj.2011.09.004

[36] Bernard HR, Killworth PD, Sailer L. Informant accuracy in social-network data V. An experimental attempt to predict actual communication from recall data. Social Science Research. 1982;11(1):30–66. 10.1016/0049-089X(82)90006-0

[37] Lazer D, Pentland AS, Adamic L, Aral S, Barabasi AL, Brewer D, et al Life in the network: the coming age of computational social science. Science (New York, NY). 2009;323(5915):721. 10.1126/science.1167742 PMC2745217 19197046

[38] Csikszentmihalyi M, Hunter J. Happiness in everyday life: The uses of experience sampling. Journal of Happiness Studies. 2003;4(2):185–199. 10.1023/A:1024409732742

[39] Thompson ER. Development and validation of an internationally reliable short-form of the positive and negative affect schedule (PANAS). Journal of cross-cultural psychology. 2007;38(2):227–242. 10.1177/0022022106297301

[40] Conner TS, Tennen H, Fleeson W, Barrett LF. Experience sampling methods: A modern idiographic approach to personality research. Social and personality psychology compass. 2009;3(3):292–313. 10.1111/j.1751-9004.2009.00170.x 19898679 PMC2773515

[41] Csikszentmihalyi M, Larson R. Validity and reliability of the Experience-Sampling Method. The Journal of nervous and mental disease. 1987;175(9):526–536. 3655778 10.1097/00005053-198709000-00004

[42] Perugini M, Di Blas L. The Big Five Marker Scales (BFMS) and the Italian AB5C taxonomy: Analyses from an emic-etic perspective. Hogrefe & Huber Publishers; 2002.

[43] Tellegen A, Waller NG. Exploring personality through test construction: Development of the Multidimensional Personality Questionnaire The Sage handbook of personality theory and assessment. 2008;2:261–292.

[44] Eagle N, Pentland AS, Lazer D. Inferring friendship network structure by using mobile phone data. Proceedings of the National Academy of Sciences. 2009;106(36):15274–15278. 10.1073/pnas.0900282106 PMC2741241 19706491

[45] Lane ND, Miluzzo E, Lu H, Peebles D, Choudhury T, Campbell AT. A survey of mobile phone sensing. Communications Magazine, IEEE. 2010;48(9):140–150. 10.1109/MCOM.2010.5560598

[46] Madan A, Cebrian M, Moturu S, Farrahi K, et al Sensing the “health state” of a community. IEEE Pervasive Computing. 2012;(4):36–45. 10.1109/MPRV.2011.79

[47] Stopczynski A, Sekara V, Sapiezynski P, Cuttone A, Madsen MM, Larsen JE, et al Measuring large-scale social networks with high resolution. PloS one. 2014;9(4):e95978 10.1371/journal.pone.0095978 24770359 PMC4000208

[48] Miller G. The smartphone psychology manifesto. Perspectives on Psychological Science. 2012;7(3):221–237. 10.1177/1745691612441215 26168460

[49] Staiano J, Lepri B, Aharony N, Pianesi F, Sebe N, Pentland A. Friends don’t lie: inferring personality traits from social network structure In: Proceedings of the 2012 ACM conference on ubiquitous computing. ACM; 2012 p. 321–330.

[50] de Montjoye YA, Quoidbach J, Robic F, Pentland AS. Predicting personality using novel mobile phone-based metrics In: Social computing, behavioral-cultural modeling and prediction. Springer; 2013 p. 48–55.

[51] Chittaranjan G, Blom J, Gatica-Perez D. Mining large-scale smartphone data for personality studies. Personal and Ubiquitous Computing. 2013;17(3):433–450. 10.1007/s00779-011-0490-1

[52] LiKamWa R, Liu Y, Lane ND, Zhong L. Moodscope: Building a mood sensor from smartphone usage patterns In: Proceeding of the 11th annual international conference on Mobile systems, applications, and services. ACM; 2013 p. 389–402.

[53] Bogomolov A, Lepri B, Ferron M, Pianesi F, Pentland AS. Daily Stress Recognition from Mobile Phone Data, Weather Conditions and Individual Traits In: Proceedings of the ACM International Conference on Multimedia. ACM; 2014 p. 477–486.

[54] Centellegher S, De Nadai M, Caraviello M, Leonardi C, Vescovi M, Ramadian Y, et al The Mobile Territorial Lab: a multilayered and dynamic view on parentsâ€^™^ daily lives. EPJ Data Science. 2016;5(1):1 10.1140/epjds/s13688-016-0064-6

[55] Nakagawa S, Schielzeth H. A general and simple method for obtaining R2 from generalized linear mixed-effects models. Methods in Ecology and Evolution. 2013;4(2):133–142. 10.1111/j.2041-210x.2012.00261.x

[56] Friedkin NE. The attitude-behavior linkage in behavioral cascades. Social Psychology Quarterly. 2010;73(2):196–213.

[57] Alshamsi A, Pianesi F, Lepri B, Pentland A, Rahwan I. Beyond Contagion: Reality Mining Reveals Complex Patterns of Social Influence. PloS one. 2015;10(8):e0135740 10.1371/journal.pone.0135740 26313449 PMC4551670

