# Supplementary material for: Network Diversity and Affect Dynamics: The Role of Personality Traits
Source: PLoS One. 2016 Apr 1;11(4):e0152358. doi: 10.1371/journal.pone.0152358 (PMC4817969; doi:10.1371/journal.pone.0152358)
Supplement: S1 File — Sections: 1 Sociometric Badges Dataset, 2 Mobile Territorial Lab Dataset, 3 Dynamic Social networks, 4 Diversity Measures, 5 Statistical Models, 6 Results. Table 1, Surveys for personality and affect states and traits in the Sociometric Badges study. Tables 2:28, statistical results. Figure 1, Descriptive statistics of the participants in the Sociometric Badges Dataset. Figure 2, Descriptive statistics of the participants in the Mobile Territorial Lab Dataset. Figure 3, The composite social network of participants in the sociometric badges dataset. Figure 4, The composite social network of participants in the MTL dataset. Figure 5, The daily social networks of the participants in the sociometric badges (face to face interaction) for days 1–15. Figure 6, The daily social networks of the participants in the sociometric badges (face to face interaction) for days 16–30. Figure 7, The daily social networks of the participants in the Living Lab Dataset (mobile phone communication) for days 1–15. Figure 8, The daily social networks of the participants in the Living Lab Dataset (mobile phone communication) for days 16–26. Figure 9, The entropy is plotted against different probabilities of a random variable. The random variable can take only two values (Bernoulli Process). Figure 10, The Gini is plotted against different probabilities of a random variable. The random variable can take only two values (Bernoulli Process). (PDF) [file pone.0152358.s001.pdf]

# *Supporting Information for:* Network Diversity and Affect Dynamics

Aamena Alshamsi[1], Fabio Pianesi[2], Bruno Lepri[2,3], Alex Pentland[3], and Iyad Rahwan[1,3,†]

[1] *Masdar Institute of Science & Technology, Abu Dhabi 54224, UAE*
[2] *Foundation Bruno Kessler, Trento, Italy*
[3] *Media Laboratory, Massachusetts Institute of Technology, Cambridge, MA*
[†] *Correspondence should be addressed to* `irahwan@acm.org`

March 11, 2016

# 1 Sociometric Badges Dataset

## 1.1 Data Collection

Through an experiment, the SocioMetric Badges Corpus tracked the activities of 52 participants [13]. The fifty two participants are employees in a research institution in Italy who volunteered to participate in the experiment for six weeks (working days are considered only). They belong to five units whereby all the employees of these units participated in the experiment along with the heads of these units. Their ages range from 23 to 53 with an average of 36 as shown in Figure 1. Forty seven participants are men (90.3%) and five are women (9%). Forty seven participants are Italian (90.3%) and five participants are from other countries (9%). Forty-six out of the fifty-three participants were researchers in computer science belonging to four research groups; the remaining six participants were part of the full-time IT support staff. Their educational level vary from high school diplomas to PhD degrees.

## 1.2 Procedure

At the beginning and at the end of the experiment, the participants filled extended surveys about (1) dispositional (stable) personality traits and (2) dispositional affective traits. These scores are considered as the dispositional factors of participants in our study. During the six weeks, participants were asked to fill three daily *experience sampling* surveys about their transient psychological states (affect) that they have experienced in the last 30 minutes. It is very unlikely that people would have experienced significantly varying affect states during such a short period of time. The surveys were triggered to be sent via email every working day at (11:00 AM, 2:00 PM and 5:00 PM). The participants were given 2.5 hours to fill the surveys. We refer to the first survey as the

morning survey, the second survey as the midday survey and the third survey as the afternoon survey.

Table 1 summarizes the types of surveys used to capture different groups of states and traits. The Big Five Marker Scale (BFMS) is widely used to assess personality scores for extraversion, agreeableness, conscientiousness, emotional stability and creativity [19]. Therefore, BFMS was used in the Sociometric Badge Corpus at the beginning and at the end of the experiment to capture personality traits of participants [13]. Similarly, Multidimensional Personality Questionnaire (MPQ) was utilized to measure dispositional affective scores of participants [20].

On the other hand, experience sampling surveys elicit transient states of affect. Questions in these surveys report participants' states which were experienced in the last 30 minutes. The short version of Positive and Negative Affect Schedule (PANAS) was used to evaluate the affective states of participants [21]. Specifically, high positive affect (HPA) was assessed using 3 items: *enthusiastic*, *interested* and *active*. High negative affect (HNA) was assessed using 3 items: *sad*, *bored* and *sluggish*. Low positive affect (LPA) was assessed using 2 items: *calm* and *relaxed* while low negative affect (LNA) was assessed using 2 items *lonely* and *isolated*.

Note that the experience sampling method has a long history and is highly reliable in measuring dynamics of psychological states within individuals [4]. For those interested in the caveats around the use of experience sampling, we also point to extensive discussions elsewhere [5].

The participants wore SocioMetric Badges every working day within the institution. These sensors are equipped with accelerometers, audio, Bluetooth and Infrared to respectively capture: body movements, prosodic speech features, co-location with other individuals and face-to-face interactions [17]. We harnessed Infrared (IR) transmissions to detect face-to-face interactions between people. In order for a badge to be detected through IR, two of them must have a direct line of sight and the receiving badge's IR must be within the transmitting badge's IR signal cone of height $h <= 1$ meter and a radius of $r <= htan\theta$, where $\theta$ = $15^{\circ}$ degrees; the infrared transmission rate (TRir) was set to 1Hz.

## 1.3 Preprocessing the surveys

The data comprise 2,254 surveys by the 52 participants. Ideally, the number of filled surveys should be 4,680 (52 participants $\times$ 3 daily surveys $\times$ 30 working days). However, participants reported absence from work 536 times [13]. This reduces the number of expected responses to 4,144. Also, it has been observed that the majority of participants used to leave the organization before 5PM on Fridays afternoon. This further reduces the number of expected responses. The response rate of filling surveys is 83.9% according to Lepri et al [13] which means that participants skip some surveys despite their availability at work. Therefore, we ended up collecting only 2,254 surveys.

We take the average of the daily scores of dynamic affect state of each participant to associate it with the daily diversity in communication that take place before the last filled survey.

**Dispositional Traits of Personality and Affect** We considered the trait scores that were reported by participants at the end of the experiment. Then, we normalized the trait scores of participants using the mean and the standard deviation. To discuss the statistical interaction between traits and social-situational factors associated with a given transition, we focused on only on participants with high scores in the trait (+1 standard deviation) and participants with low scores in the trait (-1

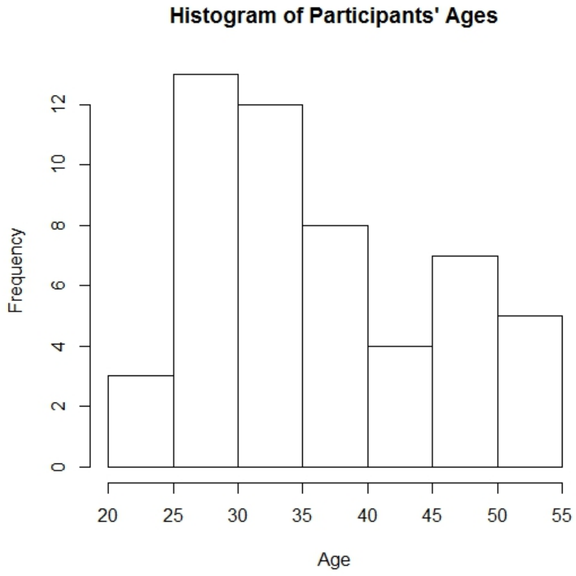


Figure 1: **Descriptive statistics of the participants in the Sociometric Badges Dataset.** The histogram displays the distribution of participants' ages.

| Group | Survey | Measurement |
|---|---|---|
| Personality | **States** | Extraversion |
| | Ten-Item Personality Inventory (TIPI) | Agreeableness |
| | | Conscientiousness |
| | **Traits** | Emotional Stability |
| | Big Five Marker Scale (BFMS) | Creativity |
| Affect | **States** | High Positive Affect |
| | Positive and Negative Affect Schedule (PANAS) | High Negative Affect |
| | **Traits** | Low Positive Affect |
| | Multidimentional Personality Questionnaire (MPQ) | Low Negative Affect |

Table 1: Surveys for personality and affect states and traits in the Sociometric Badges study

standard deviation). By using this method, we are able to know how levels of traits moderates the association between the social-situational factors and the variability in states. For example, we are interested to know how introverts respond to an increase in the diversity in social communication comparison to extroverts' response to the same increase.

# 2 Mobile Territorial Lab Dataset

## 2.1 Data Collection

The data collection was conducted inside the Mobile Territorial Lab, a joint effort between industrial (Telecom Italia and Telefonica) and academic research institutions (MIT Media Lab and FBK) launched in November 2012. It consists of a group of 150 volunteers who carry an instrumented smartphone in exchange for a monthly credit bonus of voice, SMS and data access. The sensing system installed on the smartphones logs communication events (calls and SMSs), locations, apps usage, etc. The overall goals of the MTL are to foster research on real-life behavioral analysis obtained by means of mobile devices data, and to deploy and test prototype applications in a real-life scenario. All living lab participants were recruited within the target group of young families with children, using a snowball sampling approach where existing study subject recruit future subject from among their acquaintances [8]. Upon agreeing to the terms of participation, the volunteers granted researchers legal access to their behavioral data collected by their smartphones. Volunteers retain full rights over their personal data such that they can order deletion of personal information from the secure storage servers.

Moreover, participants have the choice to participate or not in a specific study. A total of 119 volunteers from the MTL chose to participate in our data collection of daily affect states. Their ages range from 28 to 50 with an average of 39 as shown in Figure 2. Forty Three participants are men (36%) and seventy six are women (63%). 115 participants are Italian (96.6%) and participants are from other countries (3.3%). They held a variety of occupations and education levels, ranging from high school diplomas to PhD degrees. All were savvy Android users who had used the smartphones provided by the MTL since November 2012.

In our current study, we tracked the daily call social networks of 119 participant–the interlocutors could be within or outside the community of participants–in Trento, Italy for 26 days. The dynamic social networks include 3,735 transient edges and calls with total duration of 820,978 seconds. Simultaneously, daily experience sampling surveys were conducted to collect affect states .

## 2.2 Procedure

At the beginning of the study, the participants filled extended surveys about (1) dispositional (stable) personality traits and (2) dispositional affective traits. During the 26 days, participants were asked to fill daily experience sampling surveys about transient affect states that they experience. The surveys were triggered to be sent via email and via SMS every day at 8:00 PM. The participants were given 4 hours to fill the surveys.

Table 1 summarizes the types of surveys used to capture different groups of states and traits. As for the Sociometric Badges study, BFMS [19] was used at the beginning of the experiment to capture personality traits of participants. Instead, the long version of Positive and Negative Affect Schedule (PANAS) was used to measure the dispositional affective scores of participants [23].

The short version of Positive and Negative Affect Schedule (PANAS) was used to evaluate the affective daily states of participants [21]. Specifically,Positive affect (PA) was assessed using 5 items: *alert*, *inspired*, *determined*, *attentive* and *active*. Negative affect (NA) was assessed using 5

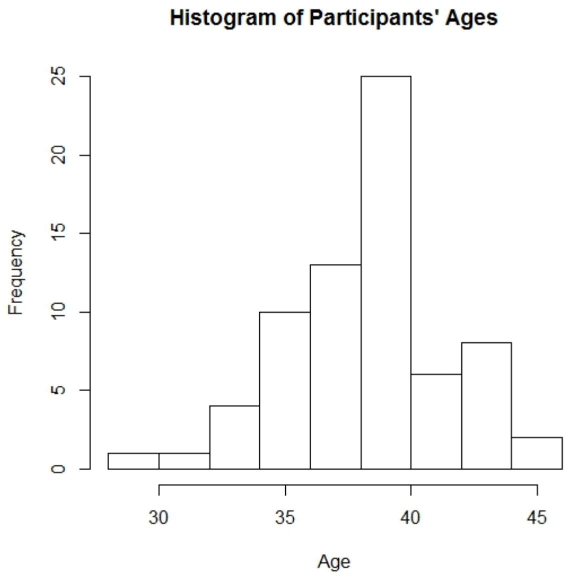


Figure 2: **Descriptive statistics of the participants in the Mobile Territorial Lab Dataset.** The histogram displays the distribution of participants' ages.

items: *upset*, *hostile*, *ashamed*, *nervous* and *afraid*.

## 2.3 Preprocessing the surveys

The data comprise 1499 surveys by the 119 participants. Ideally, the number of filled surveys should be 3,094 (119 participants $\times$ 1 daily surveys $\times$ 26 days). However, participants sometimes do not fill the daily surveys or fill it late. Because we aimed at relating the affect states to the preceding diversity in communication, the time window of the preceding communication is about twenty four hours approximately. Therefore, many surveys were not taken into consideration. Regarding the affect states, we took the exact daily reported scores.

**Dispositional Traits of Personality and Affect** We followed the same described approach for the Sociometric Badges Dataset in Section 1.3.

# 3 Dynamic Social networks

We created dynamic temporal networks of face-to-face interaction and mobile phone communication for each participant in the two datasets. For each day, we created the participant's temporal dynamic social network based on the social ties that the Infrared sensor has detected or the phone calls that the participant has received or initiated. We considered only social networks that took

place before the last filled daily survey. Figures 3 and 4 demonstrate the aggregate networks in both datasets. Figures 5, 6, 7 and 8 shows the dynamic networks in the two datasets.

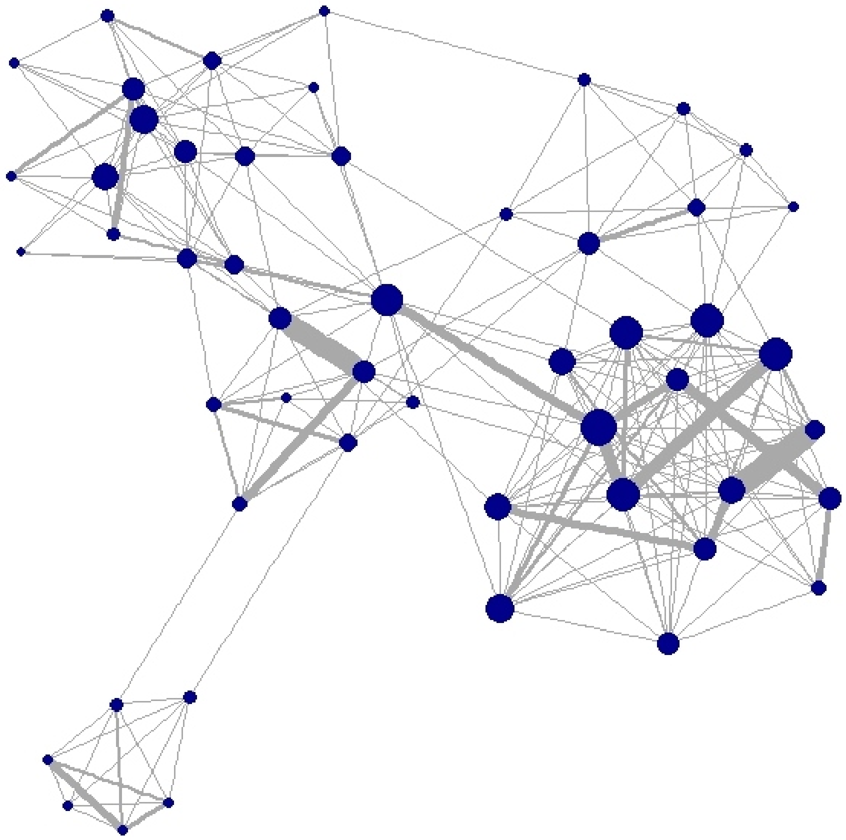

Figure 3: **The composite social network of participants in the sociometric badges dataset.** The aggregate social network is plotted showing the social ties among participants where the nodes are the participants and the edges are the IR hits detected between each pair of participants. The size of the nodes indicates their degree while the thickness of the edges is proportional number of total number of IR hits between a pair of participants. *Remarks: We considered only IR hits that are in total more than 10 to create the social ties between participants.*

# 4 Diversity Measures

We calculated the diversity in social communication using two different measures that provide different insights.

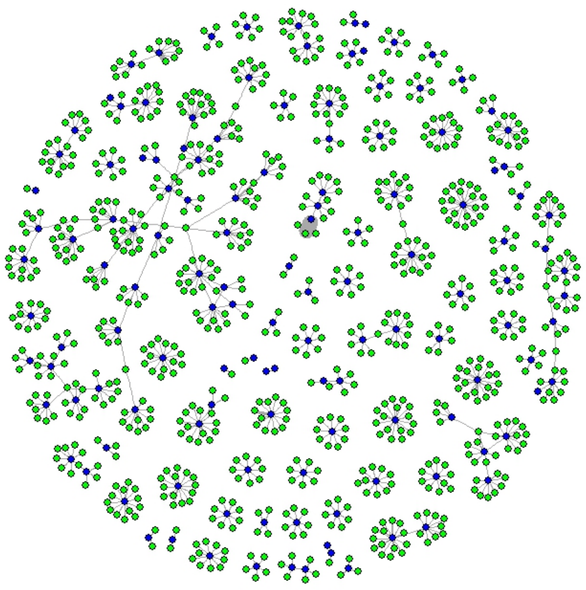

Figure 4: **The composite social network of participants in the MTL dataset** The aggregate social network is plotted showing the social ties among participants and interlocutors (other participants or non-participants) where the nodes are the participants (colored by green) or non-participants (colored by blue) and the edges are the IR hits detected between each pair of participants. *Remarks: We considered only calls with duration of 10 minutes or more in total to create the social ties between participants and their peers.*

## 4.1 Shannon's Entropy

Many researchers used Shannon's entropy to measure the diversity in communication [22, 6, 14, 12, 18]. Basically, Shannon's entropy measures the unpredictability (uncertainty) of a random variable [9]. A random variable is predictable when the probabilities of its values vary. As such, the probability of one value is higher in comparison to the probabilities of other values and therefore the outcome of the random variable becomes more predictable. The higher the inequality of the probability of the values is, the more predictable the random variable becomes.

The entropy of the random variable $X$ is calculated using the following equation where $b$ is the base of the logarithm used: $H(X) = -\sum_i P(x_i) log_b P(x_i)$. Suppose that we have only two outcomes for the random variable $X$. If the probability of one of these values is zero or one, then the entropy will be zero which means that the random variable $X$ is predictable. Figure 9 demonstrates how the entropy responds to different probabilities of one value of the random variable.

The entropy notion has been used as an index of diversity in many fields e.g. ecology [10]. It can be normalized by dividing $H(X)$ by the maximum entropy value [11] so that the value will be between of 0 and 1. In the context of social communication, the diversity is at its maximum when people split their time equally among their social networks. If we consider the unnormalized

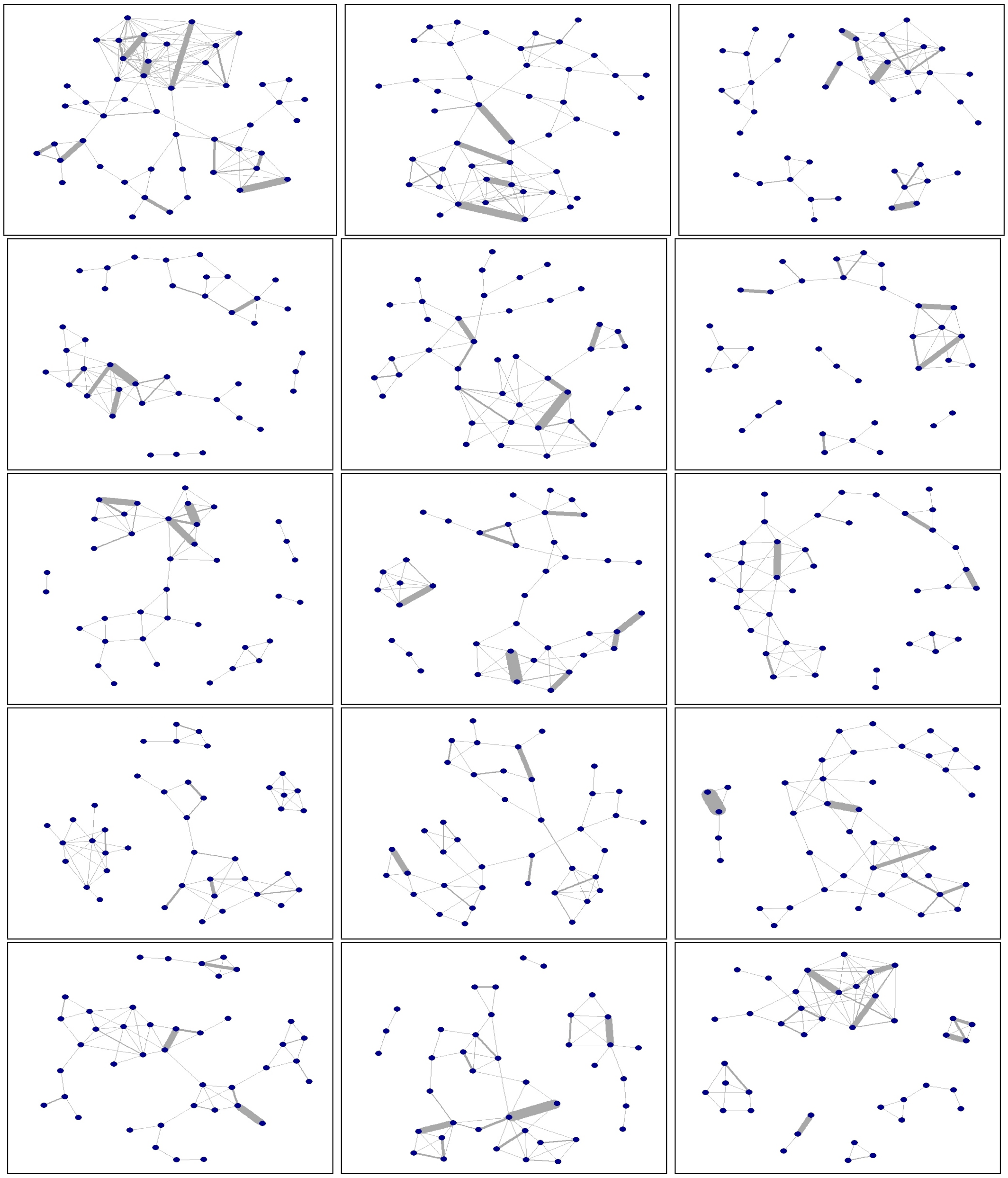

Figure 5: The daily social networks of the participants in the sociometric badges (face to face interaction) for days 1-15

version of the entropy, the value of the entropy becomes sensitive to the size of the social contacts. Splitting time evenly across four friends yields a higher entropy value in comparison to splitting time evenly across two friends.

Motivated by earlier studies and properties of Shannon's entropy, we found that Shannon's entropy is a candidate measure to denote the diversity in social communication.

First, we calculated $p_{ij} = \frac{V_{ij}}{\sum_{j}=1^{k} V_{ij}}$ where $V_{ij}$ is the communication volume between node i and j. Then, we plugged the value in the diversity equation. $D_{social}(i) = -\frac{-\sum_{j=1}^{k} p_{ij} log_2(p_{ij})}{log_2(k)}$ where

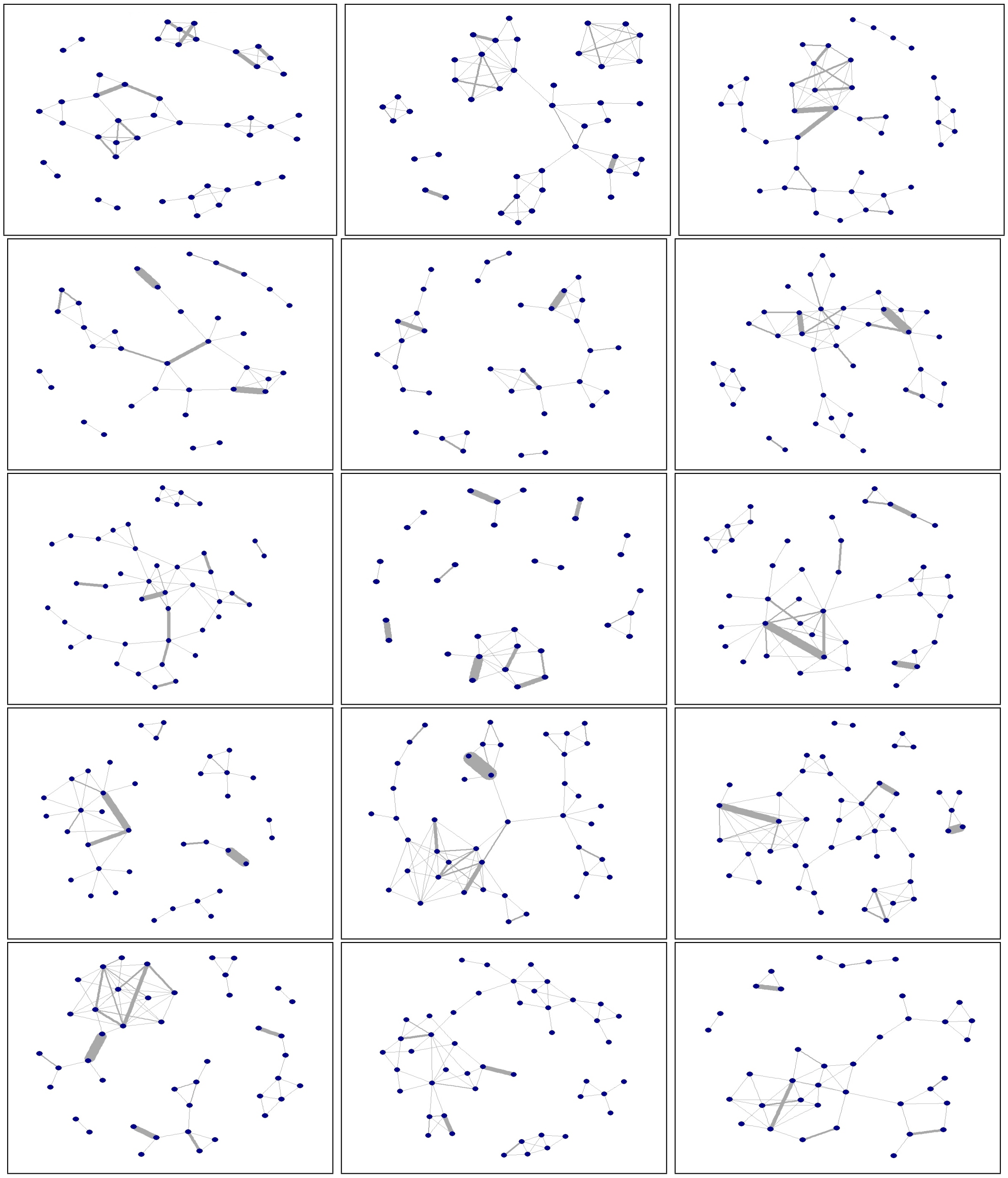

Figure 6: The daily social networks of the participants in the sociometric badges (face to face interaction) for days 16-30

k is the number of i's contacts and $p_{ij}$ is the proportion of i's total communication volume that involves j, normalized by $log_2(k)$. High entropy score means that individuals split their time more evenly between their social ties.

## 4.2 Gini Coefficient

Gini coefficient is commonly used to measure the variance in income distribution within a certain country. Basically, it captures the inequality among values of a frequency distribution. Perfect

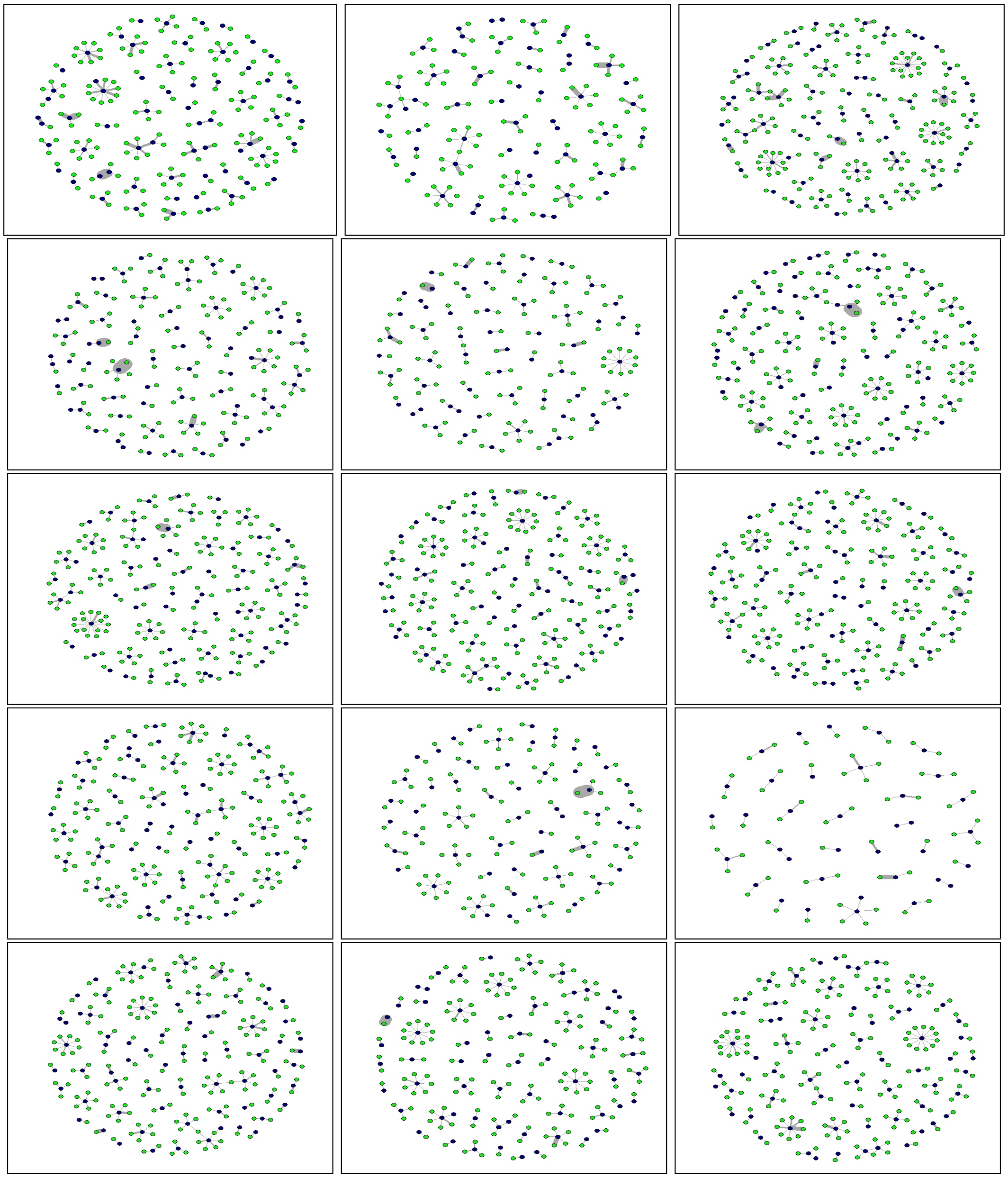

Figure 7: The daily social networks of the participants in the Living Lab Dataset (mobile phone communication) for days 1-15

inequality means that one person has the whole share of the distribution (value = 1), while perfect equality means that the shares (values) are equally distributed among people (value = 0) [7].

When a person contacts only one peer, then the inequality (Gini coefficient) will be zero. Otherwise, the distribution of time among the peers will determine the amount of the inequality. Although the Gini coefficient has not been yet used as an index to measure the diversity in communication, we assume that it is capable of capturing the diversity from a different perspective. Hence, we propose to use the Gini coefficient as another measure of diversity in social communication.

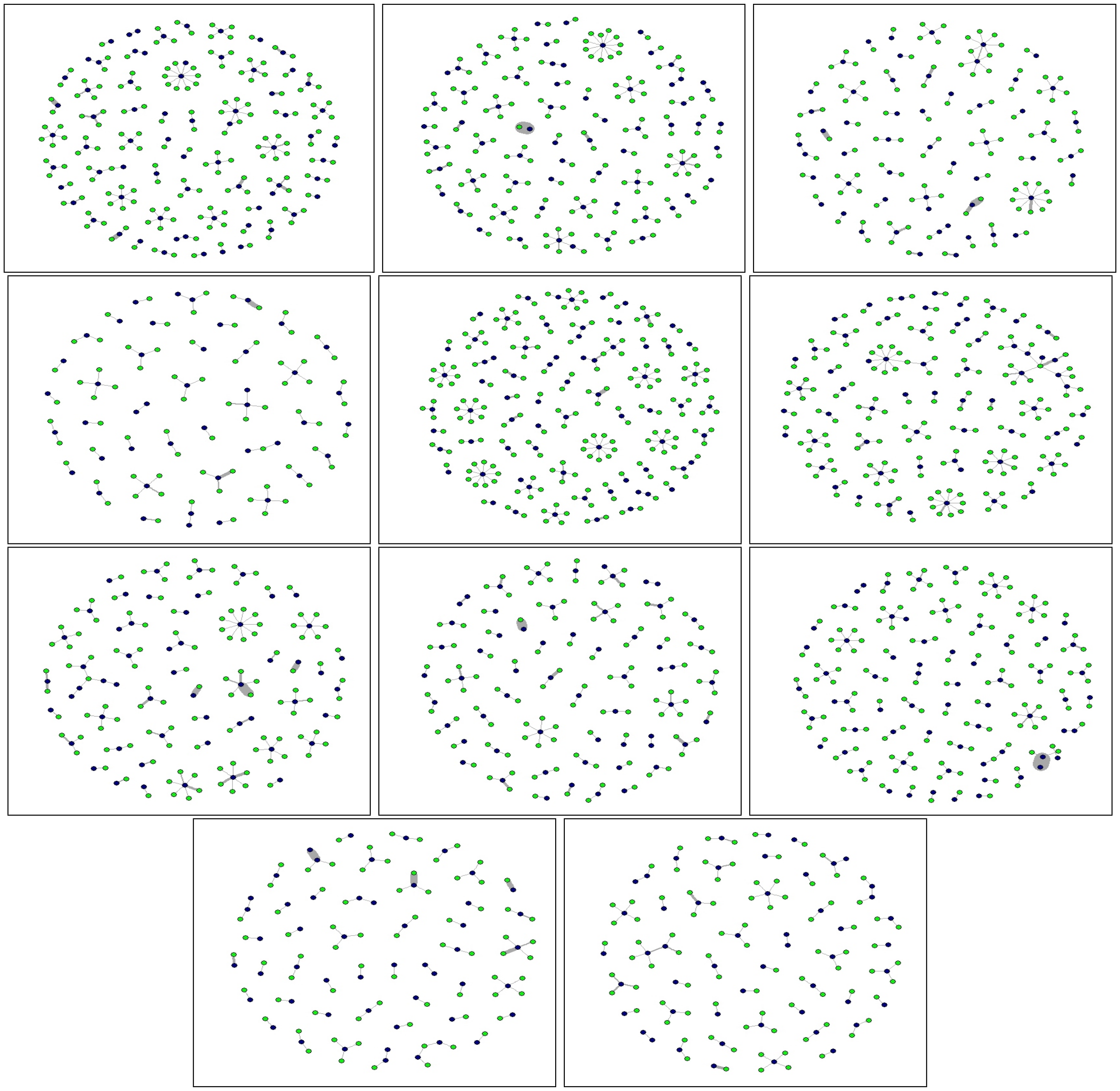

Figure 8: The daily social networks of the participants in the Living Lab Dataset (mobile phone communication) for days 16-26

# 5 Statistical Models

Both datasets are based on longitudinal studies in which daily observations are suspected to be correlated within subjects' records. Linear models such as OLS do not capture within-subject correlation. Therefore, we used generalized linear mixed models (fixed and random effects) that utilize maximum likelihood for model fitting. Fixed effects assume that the relationship between time-variant predictors and time-variant outcome variables within a specific entity (e.g. individual) could be generalized to all studies. On the other hand, random effects assume that time-invariant unobserved characteristics are specific to entities and ultimately unique to the sample that has been drawn from a larger population of entities.

In our case, we aim at understanding the relationship between time-variant diversity in social

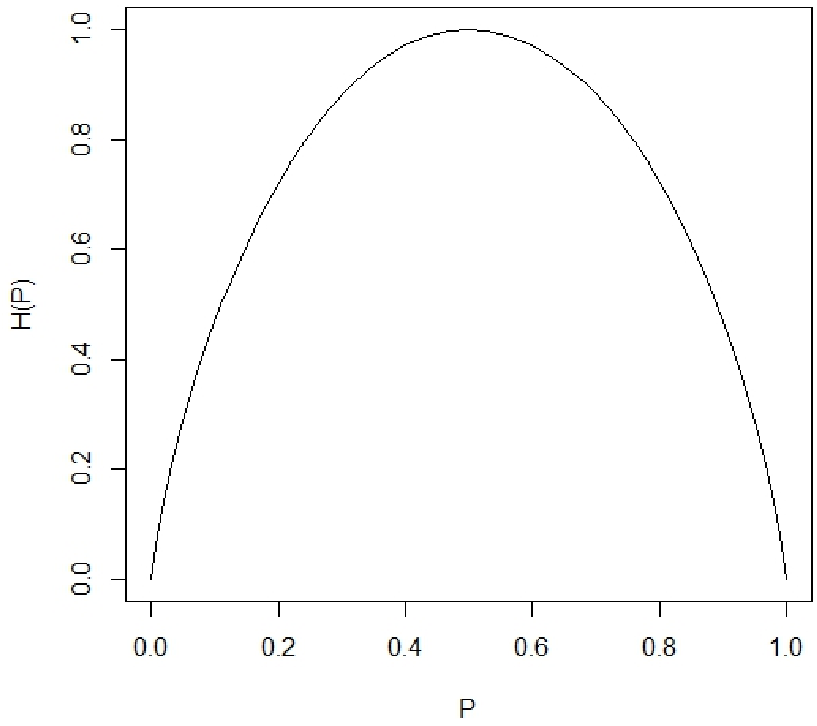


Figure 9: The entropy is plotted against different probabilities of a random variable. The random variable can take only two values (Bernoulli Process).

communication and time-variant affect states. Therefore, we calculate the fixed and random slopes of diversity in social communication in our basic model. Then, we add the individual traits and the interaction between traits and diversity measures in the complete model. Nevertheless, there could be random unobserved characteristics of individuals that are not captured in the data collection. Therefore, it is essential to control for unobserved individual characteristics by considering "individuals" as random intercepts.

## 5.1 Our Model

Our basic model consists of a diversity measure, entropy or Gini, ($X_{ij}$) that captures its fixed effect. Let $Y_{ij}$ denotes the addressed affect state, positive affect or negative affect. For each affect state and communication mode, face-to-face or phone calls, we fit the following model.

$Y_{ij} = B_0 + B_1 X_{ij} + B_2 X_{ij} + u_j + e_{ij}$. where $B_0$ is a constant (intercept), $B_1$ is the fixed effect slope of the used diversity measure, $B_2$ is the random slope of the used diversity measure, $u_j$ is the fixed intercept of individuals and $e_{ij}$ is the time-specific error.

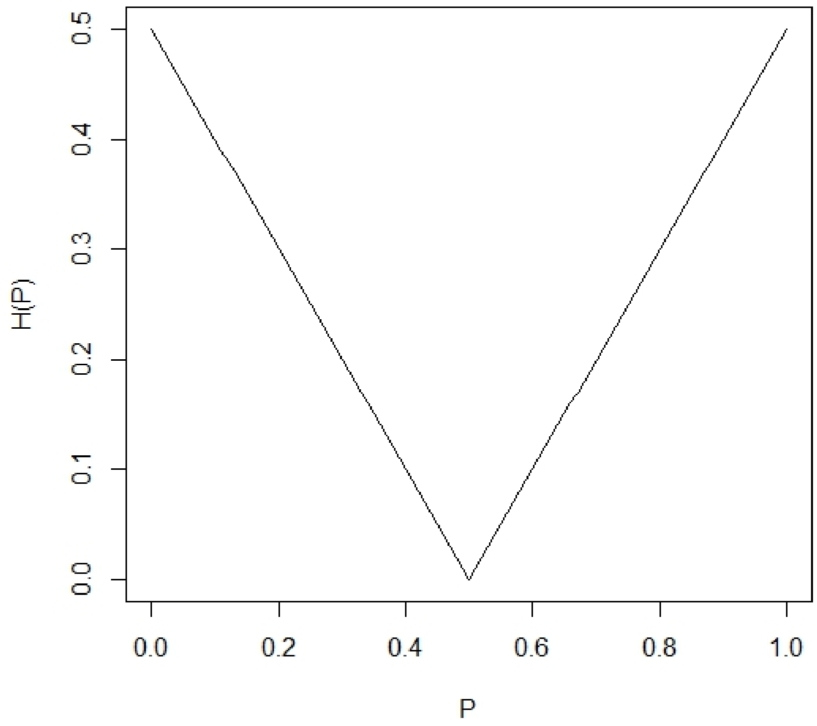


Figure 10: The Gini is plotted against different probabilities of a random variable. The random variable can take only two values (Bernoulli Process).

Our complete model contains all the variables in the basic model plus the chosen individual trait ($T_j$) and the interaction between the trait and the diversity measure ($T_j * X_{ij}$).

$Y_{ij} = B_0 + B_1 X_{ij} + B_2 X_{ij} + B_3 T_j + B_4 T_j * X_{ij} + u_j + e_{ij}$. where $B_3$ is the fixed effect of the trait and $B_4$ is the fixed effect of the interaction between the trait and the diversity measure.

## 5.2 Variance Explained

The calculation of the variance explained by models ($R^2$) is not straightforward in mixed models. Recently, Nakagawa and Schielzeth calculated two types of "variance explained" $R^2$ (1) marginal $R^2$ and (2) conditional $R^2$ [16]. Marginal $R^2$ demonstrates the variance explained by fixed effects, while the conditional $R^2$ demonstrates the variance explained by fixed and random effects. We used their method to calculate the $R^2$. In our case, the marginal $R^2$ is more relevant to quantify the absolute goodness of model fit.

### 5.3 Parameter Estimation and Model Comparison

We used the deviance test to compare each two models (basic versus complete model). The deviance, negative twice the log-likelihood, is calculated for each model. Basically, the deviance indicates quality of model fitting by maximum likelihood. The lower the deviance, the better the fit. If the reduction in the deviance caused by the complete model is statistically significant $Pr(> \chi^2) <= 0.05$, then the complete model is better than the reduced model. Otherwise, the basic (reduced) model is preferred.

Maximum likelihood ($L$) is used to estimate the parameters in linear mixed-effects. Let us assume that we have two models, $M_0$ includes only the intercept and $M_1$ includes the intercept and other fixed effects e.g. diversity measure, that were estimated using Likelihood $L_0$ and Likelihood $L_1$ respectively. From the likelihood model, we can estimate the deviance for each fitted model. In turn, the deviance (negative twice the log-likelihood) of the parameters, given the data $-2logL$ can be used to compare different models fit. Therefore, we can use the likelihood ratio test $LR = -2log\frac{L_0}{L_1} : \chi^2_p$ to compare the two models where $p$ is the number of parameters that the more complete model has. Basically, if the reduction in the deviance caused by the complete model is statistically significant $Pr(> \chi^2) <= 0.05$, then the complete model is better than the reduced model. Otherwise, the basic (reduced) model is preferred.

# 6 Results

We report our detailed results that are statistically significant. We compare between each two model (reduced and complete) in terms of the difference in deviance of the two models. If the difference in deviance is statistically significant in favor of the complete model using $\chi^2$ test, then the complete model is preferred. Otherwise, the reduced model is preferred. We also report the variance explained by each model $R^2$. Firstly, we investigate whether the daily degree distribution (number of unique social contacts) can explain the variability in daily emotions. Secondly, we present the results of diversity.

### 6.1 Degree Distribution

We found that the effect of daily degree distribution is not statistically significant in most cases. There is only one case in which the effect of degree distribution is statistically significant which is shown in Table 2.

### 6.2 Diversity

Table 2: Negative Affect in Calls: fixed intercept and random intercept (Degree Distribution)

| | *Dependent variable:* |
|---|---|
| | avg_hpa |
| Intercept | $7.6$ |
| | (p-value: $< 2e - 16$ ***) |
| Degree | $0.134$ |
| | (p-value: 0.001) |
| Observations | 912 |
| Marginal $R^2$ | 0.004 |
| Conditional $R^2$ | 0.53 |

Table 3: High Positive Affect in F2F: fixed intercept and random intercept

| | *Dependent variable:* |
|---|---|
| | avg_hpa |
| Intercept | $3.13$ |
| | (p-value: $< 2e - 16$ ***) |
| Observations | 912 |
| Marginal $R^2$ | 0 |
| Conditional $R^2$ | 0.53 |

Table 4: High Positive Affect in F2F: fixed intercept, random intercept and random slope for entropy

| | *Dependent variable:* |
|---|---|
| | avg_hpa |
| Intercept | $3.15$ |
| | (p-value: $< 2e - 16$ ***) |
| Observations | 912 |
| Marginal $R^2$ | 0 |
| Conditional $R^2$ | 0.53 |
| Difference in Deviance (Table 3) $pr(\chi^2)$ | 0.7 |

Table 5: High Positive Affect in F2F: fixed intercept, fixed effect of entropy, random intercept and random slope for entropy

| | *Dependent variable:* |
|---|---|
| | avg_hpa |
| Intercept | $3.09$ |
| | (p-value: $< 2e - 16 * **$) |
| Entropy | $0.1$ |
| | (p-value: 0.03) |
| Observations | 912 |
| Marginal $R^2$ | 0.002 |
| Conditional $R^2$ | 0.53 |
| Difference in Deviance (Table 4) $pr(\chi^2)$ | 0.03 |

Table 6: High Positive Affect in F2F: fixed intercept, random intercept and random slope for disparity

| | *Dependent variable:* |
|---|---|
| | avg_hpa |
| Intercept | $3.16$ |
| | (p-value: $< 2e - 16$ ***) |
| Observations | 912 |
| Marginal $R^2$ | 0 |
| Conditional $R^2$ | 0.54 |
| Difference in Deviance (Table 3) $pr(\chi^2)$ | 0.39 |

Table 7: High Positive Affect in F2F: fixed intercept, fixed effect of disparity, random intercept and random slope for disparity

| | *Dependent variable:* |
|---|---|
| | avg_hpa |
| Intercept | $3.2$ |
| | (p-value: $< 2e - 16 * **$) |
| Disparity | $-0.13$ |
| | (p-value: 0.055) |
| Observations | 912 |
| Marginal $R^2$ | 0.002 |
| Conditional $R^2$ | 0.54 |
| Difference in Deviance (Table 6) $pr(\chi^2)$ | 0.057 |

Table 8: High Positive Affect in F2F: fixed intercept, random intercept and random slope for gini

| | *Dependent variable:* |
|---|---|
| | avg_hpa |
| Intercept | 3.16 |
| | (p-value: $< 2e - 16$ ***) |
| Observations | 912 |
| Marginal $R^2$ | 0 |
| Conditional $R^2$ | 0.56 |
| Difference in Deviance (Table 3) $pr(\chi^2)$ | 0.0004 |

Table 9: High Positive Affect in F2F: fixed intercept, fixed effect of Gini, random intercept and random slope for Gini

| | *Dependent variable:* |
|---|---|
| | avg_hpa |
| Intercept | $3.09$ |
| | (p-value: $< 2e - 16 * **$) |
| Gini | $0.12$ |
| | (p-value: $0.18$) |
| Observations | 912 |
| Marginal $R^2$ | 0.002 |
| Conditional $R^2$ | 0.56 |
| Difference in Deviance (Table 8) $pr(\chi^2)$ | 0.18 |

Table 10: High Positive Affect in F2F: fixed intercept, fixed effect of HPA trait, random intercept and random slope for Gini

| | *Dependent variable:* |
|---|---|
| | avg_hpa |
| Intercept | $3.15$ |
| | (p-value: $< 2e - 16 * **$) |
| HPA Trait | $0.36$ |
| | (p-value: $5.52e - 10 * **$) |
| Observations | 912 |
| Marginal $R^2$ | 0.28 |
| Conditional $R^2$ | 0.54 |
| Difference in Deviance (Table 9) $pr(\chi^2)$ | $< 2.2e - 16 * **$ |

Table 11: High Positive Affect in F2F: fixed intercept, fixed effect of HPA trait, Gini and Interaction between trait and Gini, random intercept and random slope for Gini

| | *Dependent variable:* |
|---|---|
| | avg_hpa |
| Intercept | $3.09$ (p-value: $< 2e - 16 * **$) |
| Gini | $0.13$ (p-value: $0.13$) |
| HPA Trait | $0.45$ (p-value: $1.95e - 09 * **$) |
| HPA Trait*Gini | $-0.19$ (p-value: $0.03$) |
| Observations | 912 |
| Marginal $R^2$ | 0.3 |
| Conditional $R^2$ | 0.55 |
| Difference in Deviance (Table 10) $pr(\chi^2)$ | 0.03 |

Table 12: Low Negative Affect in F2F: fixed intercept and random intercept

| | *Dependent variable:* |
|---|---|
| | avg_LNA |
| Intercept | $3.13$ (p-value: $< 2e - 16 * **$) |
| Observations | 912 |
| Marginal $R^2$ | 0 |
| Conditional $R^2$ | 0.54 |

Table 13: Low Negative Affect in F2F: fixed intercept, random intercept and random slope for Gini

| | *Dependent variable:* |
|---|---|
| | avg_LNA |
| Intercept | $3.11$ (p-value: $< 2e - 16 * **$) |
| Observations | 912 |
| Marginal $R^2$ | 0 |
| Conditional $R^2$ | 0.55 |
| Difference in Deviance (Table 12) $pr(\chi^2)$ | 0.19 |

Table 14: Low Negative Affect in F2F: fixed intercept, fixed effect of Gini, random intercept and random slope for Gini

| | *Dependent variable:* |
|---|---|
| | avg_hpa |
| Intercept | 3.17 |
| | (p-value: $< 2e - 16 ***$) |
| Gini | $-0.11$ |
| | (p-value: 0.19) |
| Observations | 912 |
| Marginal $R^2$ | 0.001 |
| Conditional $R^2$ | 0.55 |
| Difference in Deviance (Table 13) $pr(\chi^2)$ | 0.19 |

Table 15: Low Negative Affect in F2F: fixed intercept, fixed effect of extroversion trait, random intercept and random slope for Gini

| | *Dependent variable:* |
|---|---|
| | avg_LNA |
| Intercept | 3.11 |
| | (p-value: $< 2e - 16 ***$) |
| Extraversion Trait | 0.066 |
| | (p-value: 0.38) |
| Observations | 912 |
| Marginal $R^2$ | 0.008 |
| Conditional $R^2$ | 0.552 |
| Difference in Deviance (Table 14) $pr(\chi^2)$ | 1 |

Table 16: Low Negative Affect in F2F: fixed intercept, fixed effect of extroversion trait, Gini and Interaction between trait and Gini, random intercept and random slope for Gini

| | *Dependent variable:* |
|---|---|
| | avg_LNA |
| Intercept | $3.18$ |
| | (p-value: $< 2e - 16 * **$) |
| Extraversion Trait | $0.17$ |
| | (p-value: 0.035) |
| Gini | $-0.12$ |
| | (p-value: 0.1) |
| Gini*Extroversion | $-0.22$ |
| | (p-value: 0.004) |
| Observations | 912 |
| Marginal $R^2$ | 0.02 |
| Conditional $R^2$ | 0.55 |
| Difference in Deviance (Table 15) $pr(\chi^2)$ | 0.005 |

Table 17: Low Negative Affect in F2F: fixed intercept, fixed effect of emotional stability trait, random intercept and random slope for Gini

| | *Dependent variable:* |
|---|---|
| | avg_LNA |
| Intercept | $3.11$ |
| | (p-value: $< 2e - 16 * **$) |
| Emotional Stability Trait | $0.15$ |
| | (p-value: 0.03) |
| Observations | 912 |
| Marginal $R^2$ | 0.04 |
| Conditional $R^2$ | 0.55 |
| Difference in Deviance (Table 14) $pr(\chi^2)$ | $< 2.2e - 16 * **$ |

Table 18: Low Negative Affect in F2F: fixed intercept, fixed effect of emotional stability trait, Gini and Interaction between trait and Gini, random intercept and random slope for Gini

| | *Dependent variable:* |
|---|---|
| | avg_LNA |
| Intercept | $3.17$ |
| | (p-value: $< 2e - 16 * **$) |
| Emotional Stability Trait | $0.21$ |
| | (p-value: $0.01$) |
| Gini | $-0.11$ |
| | (p-value: 0.17) |
| Gini*Emotional Stability | $-0.11$ |
| | (p-value: 0.18) |
| Observations | 912 |
| Marginal $R^2$ | 0.06 |
| Conditional $R^2$ | 0.55 |
| Difference in Deviance (Table 17) $pr(\chi^2)$ | 0.16 |

Table 19: Positive Affect in call dataset: fixed intercept and random intercept

| | *Dependent variable:* |
|---|---|
| | PA |
| Intercept | $18.27$ |
| | (p-value: $< 2e - 16 * **$) |
| Observations | 1499 |
| Marginal $R^2$ | 0 |
| Conditional $R^2$ | 0.52 |

Table 20: Positive Affect in call dataset: fixed intercept, random intercept and random slope for gini

| | *Dependent variable:* |
|---|---|
| | PA |
| Intercept | $18.28$ |
| | (p-value: $< 2e - 16 * **$) |
| Observations | 1499 |
| Marginal $R^2$ | 0 |
| Conditional $R^2$ | 0.53 |
| Difference in Deviance (Table 19) $pr(\chi^2)$ | 0.003 |

Table 21: Positive Affect in call dataset: fixed intercept, fixed slope of Gini, random intercept and random slope of Gini

| | *Dependent variable:* |
|---|---|
| | PA |
| Intercept | $18.26$ |
| | (p-value: $< 2e - 16$) |
| Gini | $0.05$ |
| | (p-value: $0.89$) |
| Observations | 1499 |
| Marginal $R^2$ | 0.000007 |
| Conditional $R^2$ | 0.536 |
| Difference in Deviance (Table 20) $pr(\chi^2)$ | 0.89 |

Table 22: Positive Affect in call dataset: fixed intercept, fixed slope of extroversion random intercept and random slope of Gini

| | *Dependent variable:* |
|---|---|
| | PA |
| Intercept | $18.2$ |
| | (p-value: $< 2e - 16$) |
| Extroversion | $0.67$ |
| | (p-value: 0.018) |
| Observations | 1499 |
| Marginal $R^2$ | 0.024 |
| Conditional $R^2$ | 0.54 |
| Difference in Deviance (Table 21) $pr(\chi^2)$ | $< 2.2e - 16$ |

Table 23: Positive Affect in call dataset: fixed intercept, fixed slope of extroversion, Gini and interaction between Gini and extroversion, random intercept and random slope of Gini

| | *Dependent variable:* |
|---|---|
| | PA |
| Intercept | $18.27$ |
| | (p-value: $< 2.2e-16$) |
| Gini | $-0.09$ |
| | (p-value: 0.83) |
| Extroversion | $0.23$ |
| | (p-value: 0.49) |
| Extroversion*Gini | $1.03$ |
| | (p-value: 0.02) |
| Observations | 1499 |
| Marginal $R^2$ | 0.013 |
| Conditional $R^2$ | 0.53 |
| Difference in Deviance (Table 22) $pr(\chi^2)$ | 0.06 |

Table 24: Negative Affect in call dataset: fixed intercept and random intercept

| | *Dependent variable:* |
|---|---|
| | NA |
| Intercept | $7.9$ |
| | (p-value: $< 2e-16$) |
| Observations | 1499 |
| Marginal $R^2$ | 0 |
| Conditional $R^2$ | 0.52 |

Table 25: Negative Affect in call dataset: fixed intercept, random intercept and random slope of entropy

| | *Dependent variable:* |
|---|---|
| | NA |
| Intercept | $7.9$ (p-value: 0) |
| Observations | 1499 |
| Marginal $R^2$ | 0 |
| Conditional $R^2$ | 0.52 |
| Difference in Deviance (Table 24) $pr(\chi^2)$ | 0.8 |

Table 26: Negative Affect in call dataset: fixed intercept, random intercept, random and fixed slope of entropy

| | *Dependent variable:* |
|---|---|
| | NA |
| Intercept | $7.83$ (p-value: $< 2e - 16$) |
| Entropy | $0.21$ (p-value: $0.232$) |
| Observations | 1499 |
| Marginal $R^2$ | 0.0005 |
| Conditional $R^2$ | 0.52 |
| Difference in Deviance (Table 25) $pr(\chi^2)$ | 0.23 |

# References


[1] Marc Barthélemy, Alain Barrat, Romualdo Pastor-Satorras, and Alessandro Vespignani. Characterization and modeling of weighted networks. *Physica a: Statistical mechanics and its applications*, 346(1):34–43, 2005.

[2] Marc Barthelemy, Bernard Gondran, and Eric Guichard. Spatial structure of the internet traffic. *Physica A: statistical mechanics and its applications*, 319:633–642, 2003.

[3] Stefano Boccaletti, Vito Latora, Yamir Moreno, Martin Chavez, and D-U Hwang. Complex networks: Structure and dynamics. *Physics reports*, 424(4):175–308, 2006.

Table 27: Negative Affect in call dataset: fixed intercept, random intercept, random slope of entropy and fixed slope of HNA trait

| | *Dependent variable:* |
|---|---|
| | NA |
| Intercept | $7.89$ |
| | (p-value: $< 2e-16$) |
| HNA Trait | $1.24$ |
| | (p-value: $3.33e-07$) |
| Observations | 1499 |
| Marginal $R^2$ | 0.11 |
| Conditional $R^2$ | 0.53 |
| Difference in Deviance (Table 26) $pr(\chi^2)$ | $< 2.2e-16$ |

Table 28: Negative Affect in call dataset: fixed intercept, random intercept, random slope of entropy and fixed slope of HNA trait, entropy and interaction between entropy and HNA trait

| | *Dependent variable:* |
|---|---|
| | NA |
| Intercept | $7.78$ |
| | (p-value: $< 2e-16$) |
| Entropy | $0.27$ |
| | (p-value: 0.12) |
| HNA Trait | $1.37$ |
| | (p-value: $5.67e-08$) |
| HNA*Entropy | $-0.38$ |
| | (p-value: 0.03) |
| Observations | 1499 |
| Marginal $R^2$ | 0.114 |
| Conditional $R^2$ | 0.53 |
| Difference in Deviance (Table 27) $pr(\chi^2)$ | 0.039 |


[4] Tamlin S Conner, Howard Tennen, William Fleeson, and Lisa Feldman Barrett. Experience sampling methods: A modern idiographic approach to personality research. *Social and personality psychology compass*, 3(3):292–313, 2009.

[5] Mihaly Csikszentmihalyi and Reed Larson. Validity and reliability of the experience-sampling method. *The Journal of nervous and mental disease*, 175(9):526–536, 1987.

[6] Nathan Eagle, Michael Macy, and Rob Claxton. Network diversity and economic development. *Science*, 328(5981):1029–1031, 2010.

Table 29: Negative Affect in call dataset: fixed intercept, random intercept, random slope of disparity

| | *Dependent variable:* |
|---|---|
| | NA |
| Intercept | $7.9$ |
| | (p-value: $< 2e - 16$) |
| Observations | 1499 |
| Marginal $R^2$ | 0.001 |
| Conditional $R^2$ | 0.526 |
| Difference in Deviance (Table 24) $pr(\chi^2)$ | 0.97 |

Table 30: Negative Affect in call dataset: fixed intercept, random intercept, random slope and fixed slope of disparity

| | *Dependent variable:* |
|---|---|
| | NA |
| Intercept | $8.3$ |
| | (p-value: $< 2e - 16$) |
| Disparity | $-0.52$ |
| | (p-value: 0.04) |
| Observations | 1499 |
| Marginal $R^2$ | 0.001 |
| Conditional $R^2$ | 0.53 |
| Difference in Deviance (Table 30) $pr(\chi^2)$ | 0.04 |


[7] Corrado Gini. Variabilità e mutabilità. *Reprinted in Memorie di metodologica statistica (Ed. Pizetti E, Salvemini, T). Rome: Libreria Eredi Virgilio Veschi*, 1, 1912.

[8] Leo A Goodman. Snowball sampling. *The annals of mathematical statistics*, pages 148–170, 1961.

[9] Shunsuke Ihara. *Information theory for continuous systems*, volume 2. World Scientific, 1993.

[10] Lou Jost. Entropy and diversity. *Oikos*, 113(2):363–375, 2006.

[11] Vinod Kumar. Entropic measures of manufacturing flexibility. *International Journal of Production Research*, 25(7):957–966, 1987.

Table 31: Negative Affect in call dataset: fixed intercept, random intercept, random slope of disparity and fixed slope of HNA trait

| | *Dependent variable:* |
|---|---|
| | NA |
| Intercept | $7.8$ |
| | (p-value: $< 2e-16$) |
| HNA Trait | $1.26$ |
| | (p-value: $2.34e-07$) |
| Observations | 1499 |
| Marginal $R^2$ | 0.12 |
| Conditional $R^2$ | 0.53 |
| Difference in Deviance (Table 30) $pr(\chi^2)$ | $< 2.2e-16$ |

Table 32: Negative Affect in call dataset: fixed intercept, random intercept, random slope of disparity and fixed slope of HNA trait

| | *Dependent variable:* |
|---|---|
| | NA |
| Intercept | $8.3$ |
| | (p-value: $< 2e-16$) |
| Disparity | $-0.6$ |
| | (p-value: 0.01) |
| HNA Trait | $0.79$ |
| | (p-value: 0.013) |
| HNA*Disparity | $0.57$ |
| | (p-value: 0.03) |
| Observations | 1499 |
| Marginal $R^2$ | 0.116 |
| Conditional $R^2$ | 0.53 |
| Difference in Deviance (Table 32) $pr(\chi^2)$ | 0.009 |


[12] Sang Hoon Lee, Pan-Jun Kim, Yong-Yeol Ahn, and Hawoong Jeong. Googling social interactions: Web search engine based social network construction. *PLoS One*, 5(7):e11233, 2010.

[13] Bruno Lepri, Jacopo Staiano, Giulio Rigato, Kyriaki Kalimeri, Ailbhe Finnerty, Fabio Pianesi, Nicu Sebe, and Alex Pentland. The sociometric badges corpus: A multilevel behavioral dataset for social behavior in complex organizations. In *Privacy, Security, Risk and Trust (PASSAT), 2012 International Conference on and 2012 International Confernece on Social Computing (SocialCom)*, pages 623–628. IEEE, 2012.

[14] Anmol Madan, Manuel Cebrian, David Lazer, and Alex Pentland. Social sensing for epidemiological behavior change. In *Proceedings of the 12th ACM international conference on Ubiquitous computing*, pages 291–300. ACM, 2010.

[15] Giovanna Miritello, Esteban Moro, Rubén Lara, Rocío Martínez-López, John Belchamber, Sam GB Roberts, and Robin IM Dunbar. Time as a limited resource: Communication strategy in mobile phone networks. *Social Networks*, 35(1):89–95, 2013.

[16] Shinichi Nakagawa and Holger Schielzeth. A general and simple method for obtaining r2 from generalized linear mixed-effects models. *Methods in Ecology and Evolution*, 4(2):133–142, 2013.

[17] Daniel Olguín Olguín, Benjamin N Waber, Taemie Kim, Akshay Mohan, Koji Ara, and Alex Pentland. Sensible organizations: Technology and methodology for automatically measuring organizational behavior. *Systems, Man, and Cybernetics, Part B: Cybernetics, IEEE Transactions on*, 39(1):43–55, 2009.

[18] Wei Pan, Nadav Aharony, and Alex Pentland. Fortune monitor or fortune teller: Understanding the connection between interaction patterns and financial status. In *Privacy, Security, Risk and Trust (PASSAT) and 2011 IEEE Third Inernational Conference on Social Computing (SocialCom), 2011 IEEE Third International Conference on*, pages 200–207. IEEE, 2011.

[19] Marco Perugini and L Di Blas. *The Big Five Marker Scales (BFMS) and the Italian AB5C taxonomy: Analyses from an emic-etic perspective*. Hogrefe & Huber Publishers, 2002.

[20] Auke Tellegen and Niels G Waller. Exploring personality through test construction: Development of the multidimensional personality questionnaire. *The Sage handbook of personality theory and assessment*, 2:261–292, 2008.

[21] Edmund R Thompson. Development and validation of an internationally reliable short-form of the positive and negative affect schedule (panas). *Journal of cross-cultural psychology*, 38(2):227–242, 2007.

[22] Luis M Vaquero and Manuel Cebrian. The rich club phenomenon in the classroom. *Scientific reports*, 3, 2013.

[23] David Watson, Lee A Clark, and Auke Tellegen. Development and validation of brief measures of positive and negative affect: the panas scales. *Journal of personality and social psychology*, 54(6):1063, 1988.
